# Brownian Motion Paving the Way for Molecular Translocation in Nanopores

**DOI:** 10.1002/smtd.202400042

**Published:** 2024-04-09

**Authors:** Won‐Yong Lee, Chenyu Wen, Ngan Hoang Pham, Mohammad Hadi Khaksaran, Sang‐Kwon Lee, Shi‐Li Zhang

**Affiliations:** ^1^ Division of Solid‐State Electronics Department of Electrical Engineering Uppsala University Uppsala 75103 Sweden; ^2^ Department of Bionanoscience Kavli Institute of Nanoscience Delft University of Technology Delft 2629 HZ The Netherlands; ^3^ Laboratory of Biophysics Wageningen University Wageningen 6708 WE The Netherlands; ^4^ Department of Physics Chung‐Ang University Seoul 06974 South Korea

**Keywords:** analyte trajectory, Brownian motion, electro‐osmotic flow, molecular translocation, numerical simulation, solid‐state nanopore

## Abstract

Tracing fast nanopore‐translocating analytes requires a high‐frequency measurement system that warrants a temporal resolution better than 1 µs. This constraint may practically shift the challenge from increasing the sampling bandwidth to dealing with the rapidly growing noise with frequencies typically above 10 kHz, potentially making it still uncertain if all translocation events are unambiguously captured. Here, a numerical simulation model is presented as an alternative to discern translocation events with different experimental settings including pore dimension, bias voltage, the charge state of the analyte, salt concentration, and electrolyte viscosity. The model allows for simultaneous analysis of forces exerting on a large analyte cohort along their individual trajectories; these forces are responsible for the analyte movement leading eventually to the nanopore translocation. Through tracing the analyte trajectories, the Brownian force is found to dominate the analyte movement in electrolytes until the last moment at which the electroosmotic force determines the final translocation act. The mean dwell time of analytes mimicking streptavidin decreases from ≈6 to ≈1 µs with increasing the bias voltage from ±100 to ±500 mV. The simulated translocation events qualitatively agree with the experimental data with streptavidin. The simulation model is also helpful for the design of new solid‐state nanopore sensors.

## Graphical abstract

The study investigates the translocation dynamics based on COMSOL Multiphysics mimicking the actual experiment configurations. A key development of the model is the consideration of Brownian motion in addition to the already established electroosmotic and electrophoretic forces. The results are expected to be useful not only for the analysis of experimental results but also for the design of new solid‐state nanopore sensors.

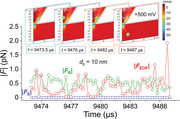

## 1 Introduction

Principally a Coulter counter^[^ ^1^ ^]^ but in nanoscale, solid‐state nanopores (SSNPs) have been explored as a single‐molecule detector operating in electrolytes for analytes such as proteins and DNA.^[^ ^2^, ^3^, ^4^, ^5^ ^]^ Apart from the superior chemical and mechanical stability of dielectric membranes in which the SSNPs are formed, the flexibility in the design of pore size, geometry, and surface charge facilitates probable exploitation of electroosmosis to manipulate molecular translocation in SSNPs.^[^ ^6^, ^7^, ^8^, ^9^ ^]^ Electroosmosis in an SSNP is primarily a consequence of the presence of surface charge on the dielectric nanopore‐sidewall;^[^ ^10^ ^]^ the surface charge induces the formation of an electrical double layer (EDL) immediately above the surface of the nanopore‐sidewall hosting counterions of the surface charge.^[^ ^11^ ^]^ Application of an external transmembrane electric voltage causes these counterions in the EDL to drift along the electric field.^[^ ^12^, ^13^ ^]^ The movement of the counterions sets the nearby water molecules in motion due to drag force (because of viscosity) thereby generating the electroosmotic flow (i.e., electroosmosis). Electroosmosis is more pronounced in smaller nanopores wherein the EDL constitutes a larger fraction of their smallest constriction; EDL is ≈1 nm in thickness for physiologically relevant electrolytes.^[^ ^10^ ^]^

Geometrically asymmetric SSNPs have been shown to rectify not only ionic current^[^ ^14^, ^15^ ^]^ but also molecular translocation.^[^ ^8^, ^9^ ^]^ Rectification refers specifically to higher currents or more frequent translocations in one direction than in the other of an SSNP. By clarifying the causal chain connecting the key physical factors and processes leading to rectification, an analytical model has recently been proposed to account for the rectified ionic current in asymmetric SSNPs as well as in their sibling nanopipettes.^[^ ^16^ ^]^ Analysis of analyte translocations in SSNPs usually focuses on the amplitude and dwell time of the spike‐like signals as well as the frequency of translocation events (FTE).^[^ ^17^, ^18^, ^19^, ^20^, ^21^ ^]^ However, the analysis is generally challenged by uncertainties in capturing the rapidly translocating analytes^[^ ^22^ ^]^ or registering distorted signals^[^ ^8^ ^]^ that may also be mistaken as noise. By assuming an amplifier with a bandwidth of 1 MHz and then a temporal resolution of 1 µs, only 6% of the total protein translocation events could be missed according to a first‐passage time distribution model.^[^ ^23^ ^]^ Thus, it is necessary to develop higher‐bandwidth measurement systems to proceed with detection regardless of the type of proteins. A 250 kHz measurement system corresponding to the minimum dwell time of ≈2.5 µs has been reported to distinguish proteins with small molecular weights below 30 kDa.^[^ ^22^ ^]^ Moreover, a solid‐state sensing platform with an extraordinary bandwidth of ≈10 MHz has been shown to be able to register a very short dwell time of ≈0.2 µs in single‐strained DNA translocation events.^[^ ^24^ ^]^ High bandwidth with the advantage of capturing signals from fast‐passing analytes of diminishing dwell time is, unfortunately, accompanied by rapidly increasing noise with frequency in the high‐frequency end above 10 kHz.^[^ ^25^, ^26^ ^]^ Hence, the actual detectable dwell time can be compromised.

Instead of striving for higher bandwidth electronics, studying the rectification of analyte translocations could be an alternative to exploring translocation behavior and nanopore design to advance analyte sensing based on SSNPs. The rectification could be represented by the FTE ratio defined as the quotient of the measured mean FTE at negative bias to that at positive bias.^[^ ^8^, ^9^ ^]^ The translocation data of streptavidin (molecular weight ≈60 kDa) and IgG_1_ (≈150 kDa), using a measurement platform with 10 kHz bandwidth, show a clear dependence of FTE ratio on the protein size relative to the smallest constriction of a truncated‐pyramidal nanopore (TPP).^[^ ^8^ ^]^ This dependency and the observation of an unprecedentedly strong rectification of DNA translocation in a bowl‐shaped nanopore (BNP)^[^ ^9^ ^]^ have been analyzed based on numerical simulation of electroosmosis. Unfortunately, this numerical approach only yields indirect information inferred from ion flow patterns and risks missing critical details pertaining to the analyte translocation itself especially considering the limited knowledge about analyte movements due to the poor temporal resolution of the measurement platform used.

The present work investigates the translocation dynamics based on COMSOL Multiphysics under conditions mimicking the actual experiment configurations as closely as possible. Studying the trajectory of each and every analyte in a large analyte cohort assuming the basic properties of streptavidin remains a focus. It is well known that for analytes of 1 µm or below in diameter, the influence of Brownian forces on their moving trajectories becomes significant. The influence is especially pronounced for analytes of a few nanometers in diameter with nanopore‐translocation times in µs or shorter using SSNPs. A key development of the model implemented on COMSOL is the consideration of Brownian force in addition to the already established electroosmotic and electrophoretic forces. Motivated by previous analytical studies based on, e.g., continuous random Gaussian field,^[^ ^27^ ^]^ we have developed a numerical simulation platform by including Brownian motion of the analytes in the presence of electrical field and fluid. The trajectories of a large analyte cohort translocating a nanopore are simultaneously monitored to yield statistically meaningful translocation behaviors. The simulation confirms some previously unknown, yet critical, subtleties with respect to analyte translocation in nanopores. The Brownian motion was previously invoked as an alternative to dielectrophoresis for particle‐particle interaction at a distance in micro‐/nano‐fluidics.^[^ ^28^ ^]^ The simulation in terms of the FTE ratio compares qualitatively well with the experiment, despite the significant difference in time scale between the simulation (10 ms) and experiment (100 s) as well as the simplifications necessary for the model development and implementation. Details revealed by the simulation are implicative for the design of new SSNP sensors.

## 2 Model Development

### 2.1 Geometrical Shape

**Figure 1:**
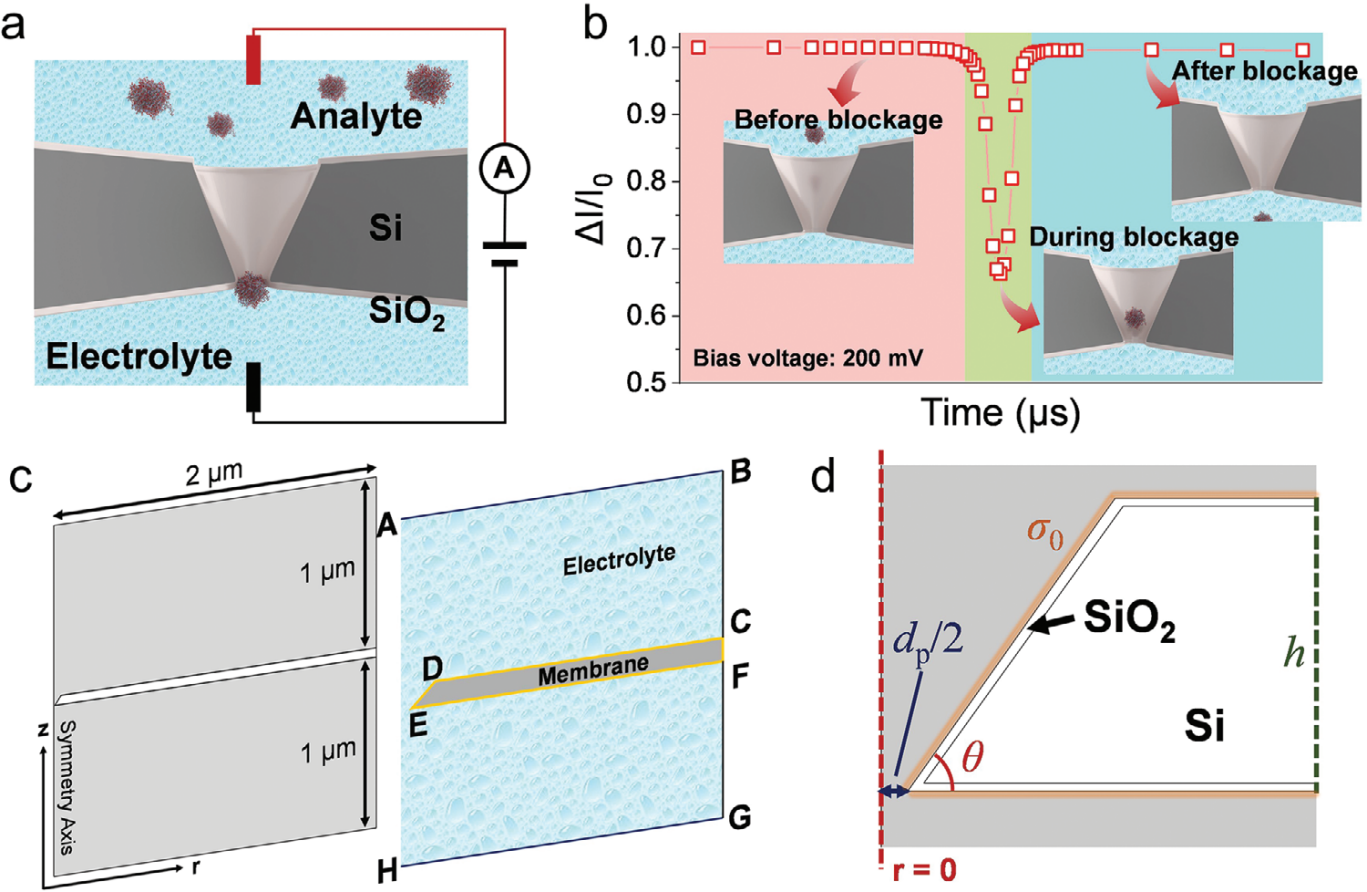
Model setup used for simulation with a truncated‐conical nanopore (TCP). a) Schematic of a two‐terminal sensing system based on the TCP of orifice diameter *d*_p_ = 10 nm biased with an externally applied voltage set to the upper reservoir relative to the ground to the lower reservoir. b) Relative ionic current drop, ∆*I*/*I*_0_, with *I*_0_ being the ionic current in the open pore state, caused by a spherical analyte of diameter 6 nm intercepting the TCP. Each point is taken from COMSOL simulation by successively changing the position of the analyte at +200 mV. c) Geometric and interface settings using the cylindrical coordinate (*r*, *z*) for a 2D axisymmetric system for COMSOL simulation. d) Enlarged geometric shape near the nanopore orifice from (c), where *σ*_0_ is the surface charge density, *h* the thickness of TCP, *d*_p_ is the diameter of the orifice, and *θ* the angle of the sloped sidewall. In all simulations, the electrolyte and *θ* are fixed to be 5x phosphate‐buffered saline (PBS) and 54.7°, respectively.

The foremost factor for performing the simulation is to set up an appropriate geometry along with material properties in the model to be implemented. In terms of geometry, the shape of truncated conical nanopores (TCPs) was adopted with similar dimensions to those of the experimental TPP. The structural details of such a TCP are illustrated in **Figure** **1**a–d to best mimic the experimental setup around TPP. To enable resource‐demanding computation, the 2D axis‐symmetric system is divided into upper and lower reservoirs relative to the conical nanopore located in the middle of the system. Each reservoir has a diameter of 2 µm and a height of 1 µm (Figure 1c). Simultaneously, the morphology of the nanopore was set to be the same as in the experiment (details can be found in Tables S1 and S2 Supporting Information).

### 2.2 Physical Properties

The nanopore region was set to Si with a native oxide layer (≈1.5 nm) and the rest to water (Figure 1d). The COMSOL material library was utilized to retrieve the representative parameters of water, i.e., density, viscosity, and relative permittivity (Table S1 Supporting Information). Models to account for the dependence of viscosity on size^[^ ^29^ ^]^ and salt concentration^[^ ^30^, ^31^ ^]^ were also incorporated. A recent study shows that the relative viscosity of nanoconfined water tends to increase with a decrease in the size of nanotubes.^[^ ^29^ ^]^ The resultant relative viscosity of ≈2.3 to ≈1.1 depends on the height (*z*) and hence on the horizontal radius of the TCP (Note S1 and Figure S1 Supporting Information). As done previously,^[^ ^8^ ^]^ the surface charge density of the naturally oxidized Si nanopore sidewall was set to −0.02 C m^−2^ to approximate the experimentally determined result of −0.016 C m^−2^. Besides, induced surface charge (ISC) due to the external electric field,^[^ ^32^ ^]^ whose density is position‐dependent along the nanopore sidewall, was also considered in the model.

Additional parameters, primarily the diffusion coefficient and concentration of Na^+^, Cl^−^, K^+^, and H_2_PO_4_^−^, in accordance with the experimentally used 5× phosphate‐buffered saline (PBS) for protein translocation studies,^[^ ^8^ ^]^ were added to facilitate the calculation of ionic movements. The diffusion coefficient was^[^ ^33^, ^34^ ^]^ 1.334 × 10^−9^, 2.032 × 10^−9^, 1.957 × 10^−9^, and 0.846 × 10^−9^ m^2^ s^−1^, whereas the concentration was set to 700, 715, 65, and 50 mM, respectively, for Na^+^, Cl^−^, K^+^, and H_2_PO_4_^−^. To examine the analyte trajectories, 319 spheres to represent the analytes adopting otherwise the size (diameter of 6 nm^[^ ^8^, ^17^ ^]^) and weight (54.31 kDa^[^ ^35^ ^]^) of streptavidin were released in each reservoir to match the protein concentration used in the experiment (84 nM).^[^ ^8^ ^]^ Detailed information about streptavidin (Protein Data Bank ID: 3RY1) was obtained from Protein Data Bank.^[^ ^35^ ^]^

### 2.3 Ionic Movement

The simulation of analyte translocations was divided into two steps: to calculate electroosmotic flow in the fluid region and to track the time evolution of input spheres. The ionic movement was jointly described by the Poisson equation,

(1) $$\nabla^{2}\Phi \;=\;-\frac{F}{\varepsilon }\sum_{i}z_{i}c_{i}$$

the Nernst–Planck equation,

(2) $$\;J_{i}=\;-D_{i}\nabla c_{i}-\frac{z_{i}F}{RT}D_{i}C_{i}\nabla \Phi +c_{i}u$$

and the Navier–Stokes equation,

(3) $$u\nabla u\;=\frac{1}{\rho }\;\left[-\nabla p+\eta \nabla^{2}u-F\left(\sum_{i}\sigma_{i}c_{i}\right)\nabla \Phi \right]$$

where, Φ, *F*, ε, *z*_i_, and *c*_i_ are, respectively, the electric potential, Faraday's constant, relative permittivity, valence, and diffusion coefficient of ion species *i* in solution. *J*_i_ is the total ion flux and *u*, ρ, *p*, and η are, respectively, the velocity, density, pressure, and viscosity of water.^[^ ^3^, ^4^ ^]^ The specific boundary conditions in Figure 1c are as follows: applied bias voltage (line A – B), ground (line G – H), axisymmetric boundary (line A – H), bulk ion concentration (line A – B and G – H), and external pressure (line A – B and G – H). The detailed boundary conditions are summarized in Table S2 (Supporting Information). Moreover, the model being developed here takes advantage of the default particle‐boundary interaction models available in COMSOL (details are summarized in Note S2, Supporting Information).

### 2.4 Analyte Translocation

The motion of a streptavidin‐like sphere can be calculated according to,

(4) $$\;\frac{d\left(m_{a}v\right)}{dt}=F_{D}\;+F_{E}+F_{B}$$

where, *m*_a_ and *v* are, respectively, the mass and velocity of the sphere, while *t* time. Exerting on the sphere, *F*_D_ is the drag force, *F*_E_ is the electrophoretic force, and *F*_B_ is the Brownian force. The force terms are expressed as follows,^[^ ^3^, ^4^, ^27^, ^36^ ^]^

(5) $$\;F_{D}=\;3\pi \eta d_{a}\;\left(u-v\right)=F_{\mathrm{EOF}}\;-3\pi \eta d_{a}v$$

(6) $$\;F_{E}=\;eZE$$

(7) $$\;F_{B}=\;\zeta \sqrt{\frac{6\pi k_{B}\eta Td_{a}}{\Delta t}}$$

where, *d*_a_, *e*, *Z*, *E*, ζ, *k*_B_, *T*, and Δ*t* are the diameter of the sphere, elementary charge, charge number, electric field, generated random vector, Boltzmann constant, the absolute temperature of the fluid, and time step taken by the solver, respectively. The electroosmotic force (*F*_EOF_ =  3πη*d*_a_ *u*) is included as part of *F*_D_. The spheres in the simulation were released at the initial time (*t* = 0 s) and the path of each sphere was computed for a total length of 10 ms at an interval of 0.25 µs, the choice of Δ*t* is detailed in Note S3 (Supporting Information) along with the physics of ζ in Note S4 (Supporting Information).

## 3 Results and Discussion

### 3.1 Electroosmotic Flow (EOF) Inside TCP

**Figure 2:**
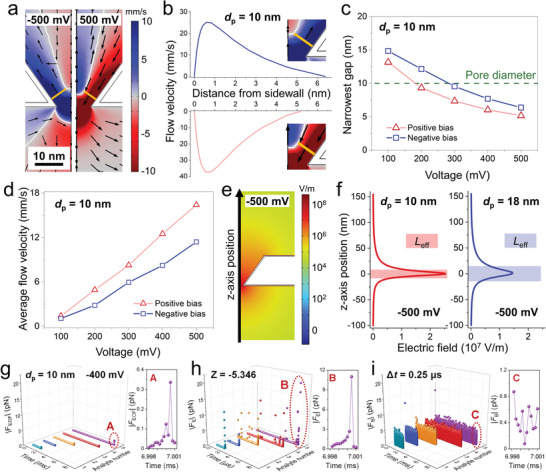
Simulated ion‐related properties for the TCP of *d*_p_ = 10 nm. a) Distribution of electroosmotic flow (EOF) at ±500 mV, with the yellow lines marking the shortest distance between the vortex and the TCP sidewall. b) Distribution of EOF velocity corresponding to the yellow lines in (a) as a function of the distance from the sidewall. c) Dependence of the narrowest gap on bias voltage for *d*_p_ = 10 nm. d) Variation of average EOF velocity in the narrowest gap with bias voltage for *d*_p_ = 10 nm. e) Distribution of electric field for *d*_p_ = 10 nm at −500 mV. f) Definition of effective transfer length, *L*_eff_, according to the distribution of electric field for *d*_p_ = 10 and 18 nm. Comparison of the magnitude of g) electroosmotic force (|*F*_EOF_|), h) electrophoretic force (|*F*_E_|), and Brownian force (|*F*_B_| with timestep (∆*t*) of 0.25 µs) exerting on analytes with a negative charge of *Z* = −5.346, all at −400 mV for *d*_p_ = 10 nm. Five analytes were randomly selected among the translocated ones to demonstrate the force evolution. Additional graphs labeled A, B, and C highlight the respective force immediately prior to the translocation event.

In the simulations to follow, a bias voltage was set to the upper reservoir in the range of −500 to +500 mV stepped by 100 mV, with respect to the ground set to the lower reservoir. The simulation results shown in **Figure** **2**a represent typical EOF patterns and vortexes formed at −500 mV (left half) and +500 mV (right half). It is well‐established by simulation^[^ ^8^ ^]^ that the forcefully drifted ionic flow in the EDL inside the nanopore is the root cause for the formation of the EOF vortex (Figure 2a; Figures S2–S4, Supporting Information). For all the results, cold color (including blue) indicates the upward flow while warm color (including red) denotes the downward flow. The straight orange lines in Figure 2a mark, in the two bias conditions, where the narrowest distance from the nanopore sidewall to the boundary of the vortex is located. On the boundary lines represented by the white curves in Figure 2a, the flow velocity in the *z*‐axis direction is zero. Inside each vortex, the EOF is in the opposite direction to that along the sidewall due to the incompressibility of electrolytes. The *z*‐direction flow velocity along the orange lines is found in Figure 2b to first rapidly increase from zero on the nanopore sidewall, to peak at ≈1 nm from the sidewall, and then to decrease to zero on the vortex boundary.

At fixed salt concentration and surface charge density, the narrowest gap between the nanopore sidewall and the vortex, defined by the length of the orange lines, is seen in Figure 2c for a 10 nm diameter (*d*_p_ = 10 nm) TCP to significantly decrease with increasing the applied bias voltage of both polarities. The results in Figure 2c conclude that the EOF vortexes move downward closer to the nanopore orifice as the bias voltage (both polarities) increases, which concurrently leads to an almost linear increase in average *z*‐direction flow velocity of the EOF at the narrowest gap displayed in Figure 2d. These trends prevail for larger pore diameters, but with weaker EOF and more distanced vortexes from the nanopore orifice (see Figures S3 and S4, Supporting Information for the simulation data of a TCP of *d*_p_ = 18 nm).

### 3.2 Electric Field Along the Nanopore Axis

In a nanopore‐electrolyte system, the applied bias voltage can affect the transport of analytes via two distinct mechanisms, i.e., electrophoresis and electroosmosis. The detection of translocating analytes is realized by analyzing temporal drops of the ionic current through a nanopore as schematically shown in Figure 1b for such a case. The current drop is intuitively caused by the temporal interception of a translocating analyte with the most resistive section of the nanopore. At least 90% of the total resistance of the nanopore‐electrolyte system results from this section confined within the so‐called effective transfer length, *L*_eff_, defined as the sum of distances where the electric field falls to *e* ^−1^ of its maximum on both sides of the nanopore along the central axis.^[^ ^37^ ^]^ The highest electric field of ≈108 V m^−1^ is found near the orifice (z = 0) of a TCP of *d*_p_ = 10 nm in Figure 2e. The electric field rapidly decreases as it moves away from the orifice resulting in *L*_eff_ = ≈16 and ≈29 nm, respectively, for TCPs of *d*_p_ = 10 and 18 nm (Figure 2f). The *L*_eff_ of TCPs are shorter than the membrane thickness, but they are ≈2–3 times the average spatial step of the randomly moving analytes (≈9.2 nm) due to *F*_B_ when adopting a timestep of 0.25 µs for calculating *F*_B_ based on Equation (7). The size of *F*_B_ predicted using this equation is obviously dependent on the Δ*t* chosen, but the reported values here are found to be in very good agreement with experimental results of the root‐mean‐square space step of particles over time.^[^ ^34^ ^]^ In terms of spatial steps (Table S3, Supporting Information) that are also determined by the Δ*t* chosen, they should be appreciably smaller than the nanopore dimension and the vortex size in order to ensure sufficient details of interactions of translocating analytes with their immediate surroundings. In this regard, the 0.25 µs timestep used appears sufficient for capturing, with certainty, a trajectory and an eventual analyte translocation in a TCP.

### 3.3 Dominant Forces for Analyte Translocation

It is plausible that a translocation event starts from an analyte migrating from the bulk electrolyte toward the nanopore predominantly via diffusion (details in Note S5 and Figures S7 and S8 of Supporting Information). The analyte, then, in the presence of an external electric field, drifts through the nanopore thereby completing the translocation process. The breakpoint between diffusion and drift can be roughly defined by *L*_eff_ in Figure 2f. The highly concentrated electric field inside *L*_eff_ results in a similarly sharp force distribution peaking inside and falling by 2–3 orders of magnitude immediately outside the nanopore.^[^ ^9^, ^32^ ^]^ Thus, the contributing forces for the drift are *F*_EOF_ and *F*_E_. Outside the nanopore including the bulk electrolyte, the analyte diffusion is determined by *F*_B_. This theoretical analysis is supported by tracking the evolutions of the forces with time and correlating them to the trajectories of five randomly selected analytes all assigned a negative charge with *Z* = −5.346 (Note S6 and Figure S9, Supporting Information). During most of the translocation time, *F*_B_ (Figure 2i) quickly alternates between ≈0.01 and 10 pN, while *F*_EOF_ (Figure 2g) and *F*_E_ (Figure 2h) stay below ≈1 fN until the analytes intercept the nanopore during which *F*_EOF_ becomes comparable to *F*_B_ and *F*_E_ is appreciably greater than *F*_B_. The consequence of *F*_E_ being the dominant force inside *L*_eff_ to, at the final step, drive the analytes through the nanopore is a total disagreement between simulation (Note S7 and Figures S10–S13a, Supporting Information) and experiment^[^ ^8^ ^]^ with respect to the translocation direction.

The *Z* value of analytes is known to be affected by the salt concentration as well as the pH of the electrolyte they are in. Employing the procedure reported in previous studies^[^ ^38^, ^39^ ^]^ led to the calculated effective *Z* of streptavidin (Protein Data Bank ID: 1SWE) to be ≈−1, −2, and −3 for 1×, 0.1×, and 0.01× PBS, respectively. The two types of streptavidin (3RY1, details in Note S6 and Figure S9, Supporting Information, and 1SWE) were calculated to carry the same *Z* by themselves according to the online protein calculator (https://www.protpi.ch/Calculator/ProteinTool), but the actual effective *Z* ought to be lower due to the Debye screening effect.^[^ ^39^ ^]^ The screening effect has also been observed when studying protein surface charge.^[^ ^40^ ^]^ By varying *Z* from −0.1, to −1, and to −5.346, the final step of analyte translocation was found to successively shift from being *F*_EOF_‐dictated to *F*_E_‐driven inside *L*_eff_ as manifested by the distinct change of the translocation direction (Figures S12 and S13b, Supporting Information). Therefore, the consideration of the high salt concentration of 5× PBS used in the experiment^[^ ^8^ ^]^ motivates our simplification of streptavidin as a charge‐neutral analyte (*Z* = 0) in the remainder of the present work.

### 3.4 Analyte Translocation

**Figure 3:**
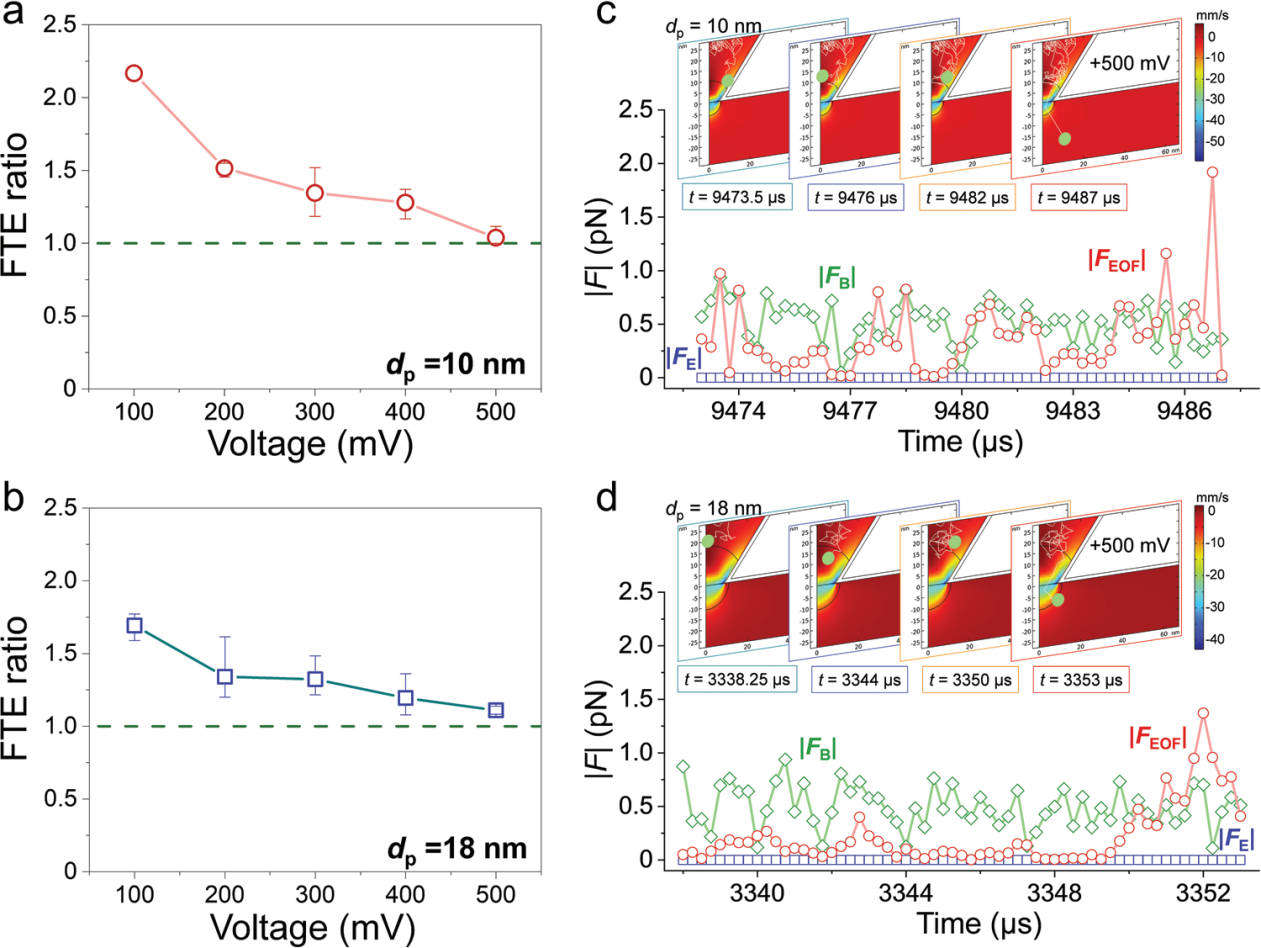
Frequency of translocation events (FTE) of charge‐neutral (*Z* = 0) analytes. Simulated FTE ratio as a function of bias voltage for TCP of a) *d*_p_ = 10 nm and b) *d*_p_ = 18 nm. Time‐dependent trajectory (upper panel) and corresponding forces (lower panel) of an analyte within its dwell time for TCP of c) *d*_p_ = 10 nm and d) *d*_p_ = 18 nm, both at +500 mV.

A successful analyte translocation can be defined with the assistance of *L*_eff_ (Note S5 and Figures S7 and S8, Supporting Information). Briefly, a translocation represents a complete process of an analyte initially (*t* = 0 s) placed in the upper (lower) reservoir traveling toward the nanopore, subsequently passing through the pore region defined by *L*_eff_, and finally landing in the lower (upper) reservoir. With this definition, the dwell time (*t*_2_ – *t*_1_, Figure S8, Supporting Information) of analytes is the duration of their presence in the region confined by *L*_eff_. The mean dwell time will be discussed momentarily below. The FTE of the charge‐neutral analytes (simulated three times at each bias voltage) was typically 20–50 per 10 ms for both *d*_p_ = 10 and 18 nm TCPs in the range of −500 to +500 mV bias voltage (Figures S14 and S15 Supporting Information). The translocations are clearly EOF‐dominated and the translocation direction is identical to that of EOF shown in Figure 2a. Those few translocations that occur in the opposite direction to the EOF can be attributed to the randomness in motion of analytes by *F*_B_ and represent a negligibly small fraction compared to the number of translocated analytes by *F*_EOF_ (Figures S14 and S15 of Supporting Information). They arise due to Equation (7) developed for the common “diffusive Brownian motion,” but it has a limitation in describing the true trajectory of an analyte since a very short timestep (≈ 0.1 ‐ 1 µs) would be required to observe a “ballistic Brownian motion.”^[^ ^41^, ^42^ ^]^ This incompatibility constitutes an inevitable error in order to apply *F*_B_ with the other forces on the same timestep (Δ*t* = 0.25 µs). Despite the relatively rare *F*_B_‐driven translocations, a number of iterative simulation calculations were carried out to improve the statistical weight. Hence, only the translocated analytes by *F*_EOF_ were registered to obtain the FTE ratio in **Figure** **3**a,b for TCPs of *d*_p_ = 10 and 18 nm, respectively. The FTE ratio for both TCPs displays a similar trend converging to unity with increasing (absolute) bias voltage in the range of 100–500 mV. This phenomenon is caused by another vortex formed in the lower reservoir when a negative bias voltage is applied and increased (Figures S2 and S3, Supporting Information). At negative bias, the analyte translocation mediated by *F*_EOF_ is determined to be in the direction from the bottom to the top. The increase in both dimension and flow velocity of this lower vortex with the amplitude of negative bias voltage narrows the gap between the nanopore and the vortex, thereby, making it increasingly difficult for the analytes to enter the nanopore from below. The observation of the simulated FTE ratios converging to unity at high bias voltages for both TCPs of *d*_p_ = 10 and 18 nm is in qualitative agreement with the experimental data.^[^ ^8^ ^]^ However, clear quantitative discrepancies remain to be addressed. The difficulty in fully replicating real‐world experiments in simulation is an outstanding challenge. For instance, changes in the salt concentration of the electrolyte will not only alter the magnitude of *F*_EOF_ but will also affect the effective net charge of the analytes and should be considered for both *F*_EOF_ and *F*_E_, while the effect of partially varying viscosity within the nanopore should be carefully incorporated into the simulation as it will affect *F*_EOF_, *F*_E_, and *F*_B_. Approximating the experimental TPP using a TCP to facilitate the time‐demanding simulation may have overlooked some subtle yet critical details such as the variation of viscosity with dimension (Note S1 and Figure S1, Supporting Information). The qualitative agreement is, nonetheless, considered sufficient for employing the model to perform the analysis of the forces acting on the analytes.

The trajectory and corresponding forces during the dwell time of a randomly selected analyte are depicted in Figure 3c,d for the TCPs of *d*_p_ = 10 and 18 nm at a fixed bias voltage of +500 mV, respectively, to elucidate the important details at the final step of a translocation (along with similar data but at −500 mV in Figure S16, Supporting Information). In both cases, *F*_EOF_ plays a decisive role whereas *F*_B_ also has a significant impact, and *F*_E_ = 0 due to charge‐neutral analytes assumed. Along the trajectory from the upper to lower boundaries of *L*_eff_, *F*_B_ dominates over *F*_EOF_ and *F*_E_ during most of the dwell time with a random movement of the analyte inside the region between the boundaries of *L*_eff_. In other words, the analyte will largely move randomly for *F*_B_ > *F*_EOF_ while maintaining the directional movement by *F*_EOF_; but it will follow the streamline of EOF flow with slight wobbling for *F*_B_ < *F*_EOF_. While the magnitude of *F*_B_, |*F*_B_|, fluctuates in the range of 0.05–0.94 pN (Figure 3c,d), the analyte can often reach a spot at which the magnitude of *F*_EOF_, |*F*_EOF_|, exceeds |*F*_B_|. Finally, the analyte completes the translocation by the dominant *F*_EOF_ at ≈1.5 pN. This trend becomes more pronounced for larger *d*_p_ (Figure 3d), as *F*_EOF_ weakens rapidly upon its movement away from the sidewall (Figure 2b).

**Figure 4:**
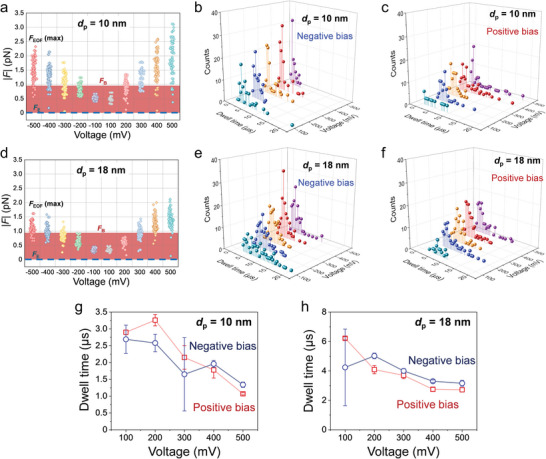
Statistical analysis of force and dwell time for translocated analytes (*Z* = 0). Comparison of the maximum force magnitude of *F*_EOF_, *F*_EOF_ (max), to the magnitude ranges of *F*_E_ and *F*_B_ exerting on individual analytes at bias voltages from ±100 to ±500 mV for TCP of a) *d*_p_ = 10 nm and d) *d*_p_ = 18 nm. Distribution of dwell time for translocated analytes at bias voltages from +100 to +500 mV for b,c) *d*_p_ = 10 nm and e,f) *d*_p_ = 18 nm. Extracted mean dwell time as a function of bias voltage for g) *d*_p_ = 10 nm and h) *d*_p_ = 18 nm. The mean dwell time for the case of *d*_p_ = 10 nm at +100 mV was taken from the geometric mean of the histogram, while it was determined by Gaussian fitting for the rest.

The ability to trace the trajectory of individual randomly moving analytes (**Figure** **4**; Figures S7 and S8, Supporting Information) in combination with the definition of dwell time based on the concept of *L*_eff_ allows for the exploration of translocation characteristics in depth. First, how the maximum magnitude of *F*_EOF_, |*F*_EOF_ (max)|, varies with bias voltage within the dwell time is shown in Figure 4a,d for analytes translocating TCPs of *d*_p_ = 10 and 18 nm, respectively. Whereas |*F*_B_| randomly fluctuates in the range of 30 fN–0.96 pN (filled area in red) regardless of the bias voltage, a substantial increase in |*F*_EOF_ (max)| from ≈0.3 to ≈1.9 pN (obtained from the average value of translocated analytes) are proportionally dependent on the bias voltage. The relationship between |*F*_B_| and |*F*_EOF_ (max)| is naturally associated with the distribution of dwell time, as shown in Figure 4b,c,e,f for *d*_p_ = 10 and 18 nm, respectively. The larger distribution of |*F*_EOF_ (max)| at positive bias than that at negative bias in Figure 4a,d leads to a wider distribution of dwell time in Figure 4c,f. These statistical results support the presence of an internal vortex as the cause responsible for the increase in dwell time because the EOF inside the vortex is in the opposite direction to the EOF along the nanopore sidewall (Figure 2a). The distribution of dwell time is Gaussian for all cases but the one with *d*_p_ = 10 nm at +100 mV (Figures S17–S20, Supporting Information), which leads to the extraction of mean dwell time summarized in Figure 4g,h. For both cases of *d*_p_ = 10 and 18 nm, the mean dwell time tends to decrease with increasing bias voltage, conforming with the anticipated impact of *F*_EOF_ whose magnitude is seen to increase with bias voltage in Figure 4a,d. At ±100 mV, the analytes experience a lower EOF impact than at higher voltages because |*F*_EOF_ (max)| always lies in the range of |*F*_B_|, implying that the analytes retain the directionality by *F*_EOF_ but have a more random behavior than otherwise at higher bias voltage. Hence, the analytes have a longer mean dwell time from ≈3 to ≈6 µs. Above ±100 mV, the mean dwell time decreases and it becomes ≈1 (*d*_p_ = 10 nm) and ≈2 µs (*d*_p_ = 18 nm) at ±500 mV. Finally, the simulated mean dwell times are also comparable to experimental results for other types of proteins,^[^ ^22^, ^23^, ^43^ ^]^ implying that our model can be helpful in exploring the experimentally inaccessible details due, for instance, to bandwidth limitations as well as in developing SSNPs with new design such as TPP and BNP.^[^ ^22^, ^23^, ^43^ ^]^

## 4 Conclusion

We have developed a model based on numerical simulations to account for our earlier experimental observations of rectified protein translocations in geometrically asymmetric nanopores. A key feature of the model is the consideration of Brownian force, in addition to electrophoretic and electroosmotic forces incorporated in numerous previous studies of ionic transport in nanopores. Extensive evaluation of various boundary conditions and modules available on the commercial simulation platform used, COMSOL Multiphysics, as well as of additional models is necessary and has led to a successful implementation of the model for numerical simulations. In the large parameter space considered, electrolyte viscosity is found to be an influential factor in analyte translocation behavior and outcome. The simulation predicts the electroosmotic force being determinant at the last moment of translocation for neutral analytes, insomuch as the electrophoretic force being determinant for charged ones. However, the pathfinding and statistical analysis of the analyte movements confirm that Brownian force on the analytes plays the dominant role all the way until the last moment of their translocation. The simulated dwell times show comparable values to the experimentally determined results for proteins with similar molecular weights. Our results are expected to be useful not only for the analysis of experimental results but also for the design of new solid‐state nanopore sensors.

## Conflict of Interest

The authors declare no conflict of interest.

## Author Contributions

W.‐Y.L. and C.W. contributed equally to this work. S.‐L.Z. conceived the idea and supervised the project. W.‐Y.L. performed all simulation work supported by close follow‐ups of S.‐L.Z. and C.W. N.H.P, M.H.K, and S.‐K.L. contributed with discussion. W.‐Y.L. and S.‐L.Z. wrote the manuscript. All authors commented on the manuscript.

## Supporting information

Supporting Information

## Data Availability

The data that support the findings of this study are available from the corresponding author upon reasonable request.
