# Supplementary material for: Brownian Motion Paving the Way for Molecular Translocation in Nanopores
Source: Small Methods. 2024 Apr 9;8(12):2400042. doi: 10.1002/smtd.202400042 (PMC11672184; doi:10.1002/smtd.202400042)
Supplement: Supplementary file 1 — Supporting Information [file SMTD-8-2400042-s001.pdf]

small methods

Supporting Information

for *Small Methods*, DOI 10.1002/smtd.202400042

Brownian Motion Paving the Way for Molecular Translocation in Nanopores

*Won-Yong Lee, Chenyu Wen, Ngan Hoang Pham, Mohammad Hadi Khaksaran, Sang-Kwon Lee and Shi-Li Zhang**

Supporting Information

Brownian motion paving the way for molecular translocation in nanopores

Won-Yong Lee,[1‡] Chenyu Wen,[2,3‡] Ngan Hoang Pham,[1] Mohammad Hadi Khaksaran,[1] Sang-Kwon Lee,[4] and Shi-Li Zhang[1]*

[1] *Division of Solid-State Electronics, Department of Electrical Engineering, Uppsala University, Uppsala 75103, Sweden*

[2] *NanoDynamicsLab, Laboratory of Biophysics, Wageningen University, Wageningen 6708 WE, The Netherlands*

[3] *Department of Bionanoscience, Kavli Institute of Nanoscience, Delft University of Technology, Delft 2629 HZ, The Netherlands*

[4] *Department of Physics, Chung-Ang University, Seoul 06974, Republic of Korea*

* Address correspondence to shili.zhang@angstrom.uu.se

Table of Contents:

Table S1: Material parameters for COMSOL simulation.
Table S2: Boundary conditions for COMSOL simulation.
Table S3: Brownian motion of nanoparticles with similar physical properties of streptavidin in water with respect to the set timestep.
Note 1: Factors causing discrepancies in simulated data from real-world results.
Note 2: Particle-wall interaction in COMSOL simulation.
Note 3: Brownian force equation in COMSOL simulation.
Note 4: Random vector generator for evaluating the Brownian force.
Note 5: Definition of translocation in COMSOL simulation.
Note 6: Properties of streptavidin used in COMSOL simulation.
Note 7: Effective net charge setting in COMSOL simulation.
Figure S1: Simulation results for the consideration of local viscosity variations inside a TCP.
Figure S2 – S3: Distribution of EOF velocity for a TCP of d_p = 10 and 18 nm.
Figure S4: Details of EOF flow in a TCP of d_p = 18 nm with respect to the bias voltages.
Figure S5 – S6: Tracking 20 analytes over time by Brownian force with 'randomnormal' respectively 'random' function as a random vector generator.
Figure S7: Definition of zones (1 – 4) in the model for registering translocations.
Figure S8: Determination of the dwell time of successful translocations in COMSOL simulation.
Figure S9: Calculated titration curve of streptavidin (3RY1) as a function of pH value.
Figure S10: Trajectory plots of five analytes corresponding to Figure 2g – i.
Figure S11: Time-dependent analyte distribution for d_p = 10 with negative charged analytes.
Figure S12: Analyte distribution (t = 10 ms) for d_p = 10 according to the effective net charge.
Figure S13: Summary of translocated analytes conducted in Figure S9 and S10.
Figure S14 – S15: Summary of translocated charge neutral analytes for d_p = 10 respectively 18 nm as a function of bias voltage.
Figure S16: Time-dependent trajectory and forces of an analyte for negative bias; d_p = 10 and 18 nm.
Figure S17 – S20: Histograms of simulated dwell time for d_p = 10 and 18 nm as according to the bias voltage.

Configuration of COMSOL Simulation

Table S1. Material parameters for COMSOL simulation.

Material	Density [kg/m^3]	Dynamic viscosity [Pa·s]	Relative permittivity	Diffusion coefficient [10^{-9} m^2/s]
Si	2.330×10^3 *	-	11.7 [1]	-
SiO_2	2.649×10^3 *	-	3.9 [2]	-
Water	0.996×10^3 *	8.5×10^{-4} *	80 [2]	-
Na^+	-	-	-	1.334 [3]
Cl^-	-	-	-	2.032 [3]
K^+	-	-	-	1.957 [3]
$H_2PO_4^-$	-	-	-	0.846 [4]

*Default values in the COMSOL Material library.

Table S2. Boundary conditions for COMSOL simulation.

Boundary	Length [nm]	Boundary equations		
		Poisson	Nernst-Planck	Navier-Stokes
AB	2000	$V = V_0$	$c_i = c_{0,i}$	Inlet, $p = 0$
BC	1000	$\boldsymbol{n}\cdot\boldsymbol{D} = 0$	$-\ \boldsymbol{n}\cdot\boldsymbol{J}_i = 0$	No slip, $v = 0$
CD	1956 (for d_p = 10 nm)<br>1952 (for d_p = 18 nm)	$\sigma = \sigma_0$	$-\ \boldsymbol{n}\cdot\boldsymbol{J}_i = 0$	No slip, $v = 0$
DE	67 (for both d_p = 10 and 18 nm)	$\sigma = \sigma_0$	$-\ \boldsymbol{n}\cdot\boldsymbol{J}_i = 0$	No slip, $v = 0$
EF	1995 (for d_p = 10 nm)<br>1991 (for d_p = 18 nm)	$\sigma = \sigma_0$	$-\ \boldsymbol{n}\cdot\boldsymbol{J}_i = 0$	No slip, $v = 0$
FG	1000	$\boldsymbol{n}\cdot\boldsymbol{D} = 0$	$-\ \boldsymbol{n}\cdot\boldsymbol{J}_i = 0$	No slip, $v = 0$
GH	2000	$V = 0$ (ground)	$c_i = c_{0,i}$	Outlet, $p = 0$
CF	55	$\boldsymbol{n}\cdot\boldsymbol{D} = 0$	-	-
AH	2055	Axial symmetry line		

V = electrical potential; V_0 = applied bias voltage; c_i = concentration of ion species i; $c_{0,i}$ = applied concentration of ion species i at the interfaces; p = pressure applied to the fluid; $\boldsymbol{n}$ = normal direction vector; $\boldsymbol{D}$ = electric displacement field; $\boldsymbol{J}_i$ = ionic flux of ion species i; v = water flow velocity; σ = surface charge density; σ_0 = applied value for surface charge density.

Note 1. Factors causing discrepancies in real-world results

The results of the current COMSOL simulation model with TCP (see Figure 3a,b) are qualitatively consistent with our experimental results with TPP[5] in the sense that the FTE ratios for nanopores with d_p = 10 and 18 nm tend to converge to unity with increasing bias voltage from 100 to 500 mV (see Figure 4d in Ref. 5). However, there are quantitative differences that can be caused by variations in the experimental environment and subtle yet critical details in the simulation settings. First, a TCP differs from a TPP in their structures. Although the two structures are very similar, they have different L_{eff}[6] partly resulting from the difference in distribution of electric field inside the nanopores, which directly affects the EOF velocity. Second, the local variation of electrolyte material properties within the nanopore can affect the FTE ratio. The capillary effect becomes more pronounced for dimensionally more constricted (e.g., narrower) nanopores. A recent study shows that the relative viscosity of nanoconfined water tends to increase with decreasing the size of nanotubes.[7] Figure S1 shows simulation results that take this effect into account. In Figure S1a, this model is exclusively applied to the interior of the nanopore, using the following equation;

$$\frac{\eta}{\eta_0} = 10.14\left(d_p\right)^{-0.9107} + 1.035 = 10.14(5 + \cot(54.7°) \cdot z)^{-0.9107} + 1.035 \quad \text{(S1)}$$

where η is the viscosity of the electrolyte, η_0 the bulk viscosity of pure water, and z the height inside the nanopore region measuring from the pore orifice upwards ($0 \le z \le 55$ nm). The resultant relative viscosity of ~2.3 to ~1.1 depending on z and hence on the horizontal radius of the TCP is shown Figure S1b. The influence of this capillary effect is an average reduction of the simulated FTE ratio by ~50% for d_p = 10 nm (Figure S1c) and ~20% for d_p = 18 nm (Figure S1d). These results indicate that the nanopore size effect constitutes a major contributor to the differences between experimental and simulation results regarding the FTE ratio. Certainly, it is insufficient to account for the experimentally observed FTE ratio being smaller than unity when analytes appreciably smaller than the nanopore size translocate the TPP. Other factors to consider include changes in ion density and viscosity resulting from ion migration caused by the electric field.[8, 9] Narrower pores bring on an additional ion gradient, which may result in an increased FTE ratio. However, this factor is subject to constant fluctuations in real-time, requiring much more sophisticated models implementable in numerical simulations.

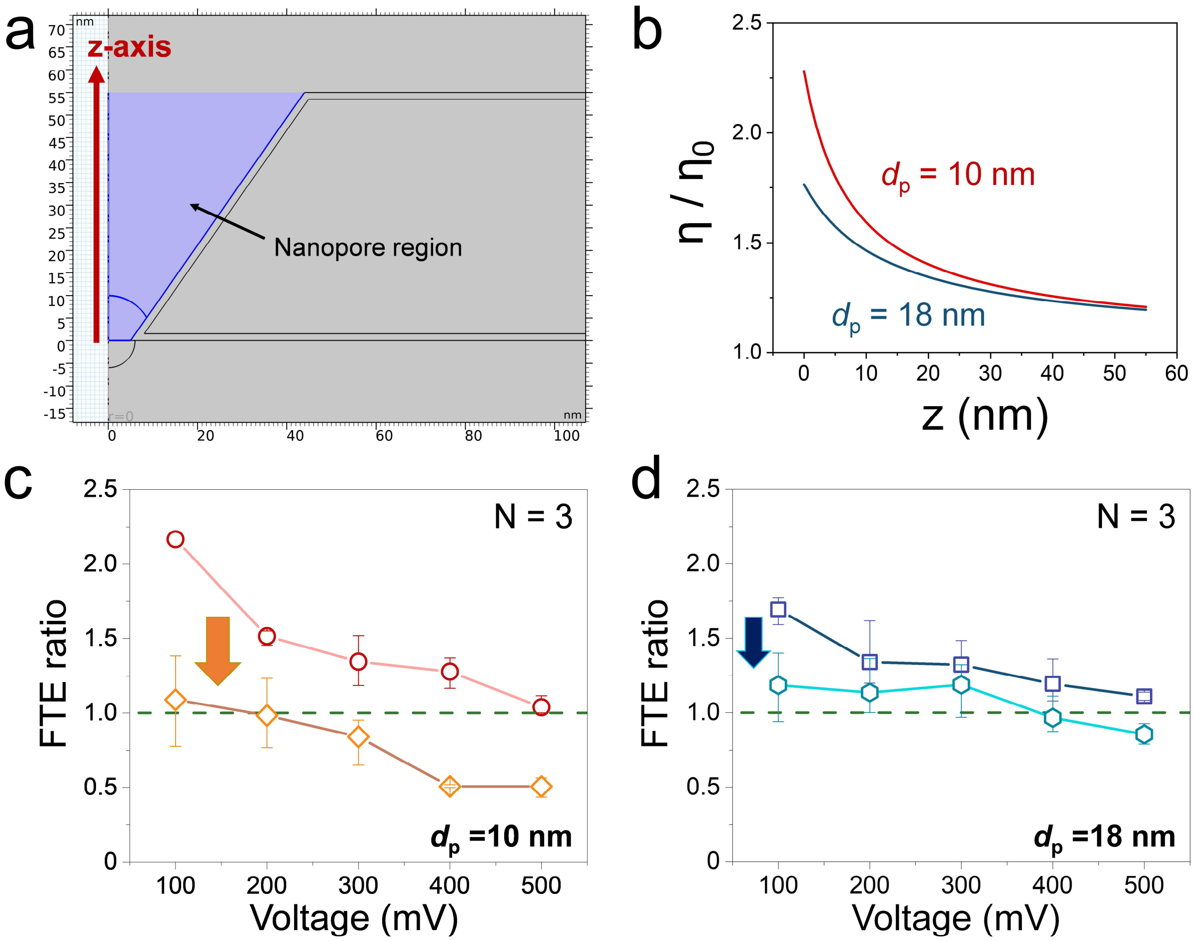

Figure S1. Simulation results by considering the effect of local viscosity variations within the nanopore. (a) Model setup with the electrolyte region inside the nanopore. The truncated cone has a different diameter along its height, *z*. (b) Relative viscosity as a function of *z* inside the nanopore region. The relative viscosity (η/η_0) indicates the degree to which it has increased compared to that of bulk water (η_0). Results of reduced FTE ratio with increasing relative viscosity in simulation for TCP of (c) d_p = 10 nm and (d) d_p = 18 nm, with 'N' referring to the iteration number of each simulation.

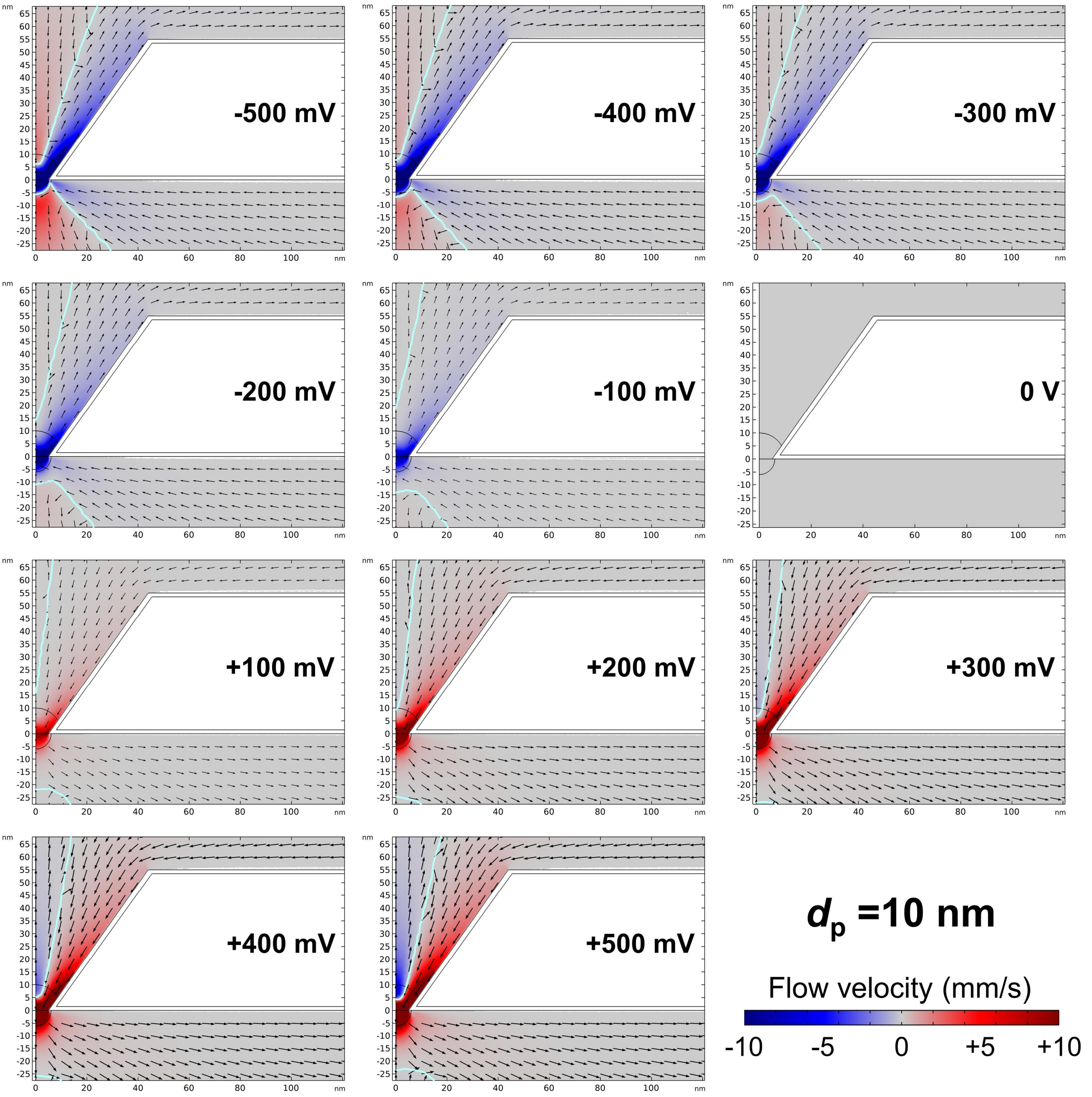

Figure S2. Distribution of EOF velocity for a TCP of d_p = 10 nm in the range of applied bias voltage from -500 mV to +500 mV. Black curves immediately above and below the nanopore orifice mark where L_{eff} extends to for each case, while the cyan curves indicate the boundaries of the vortices.

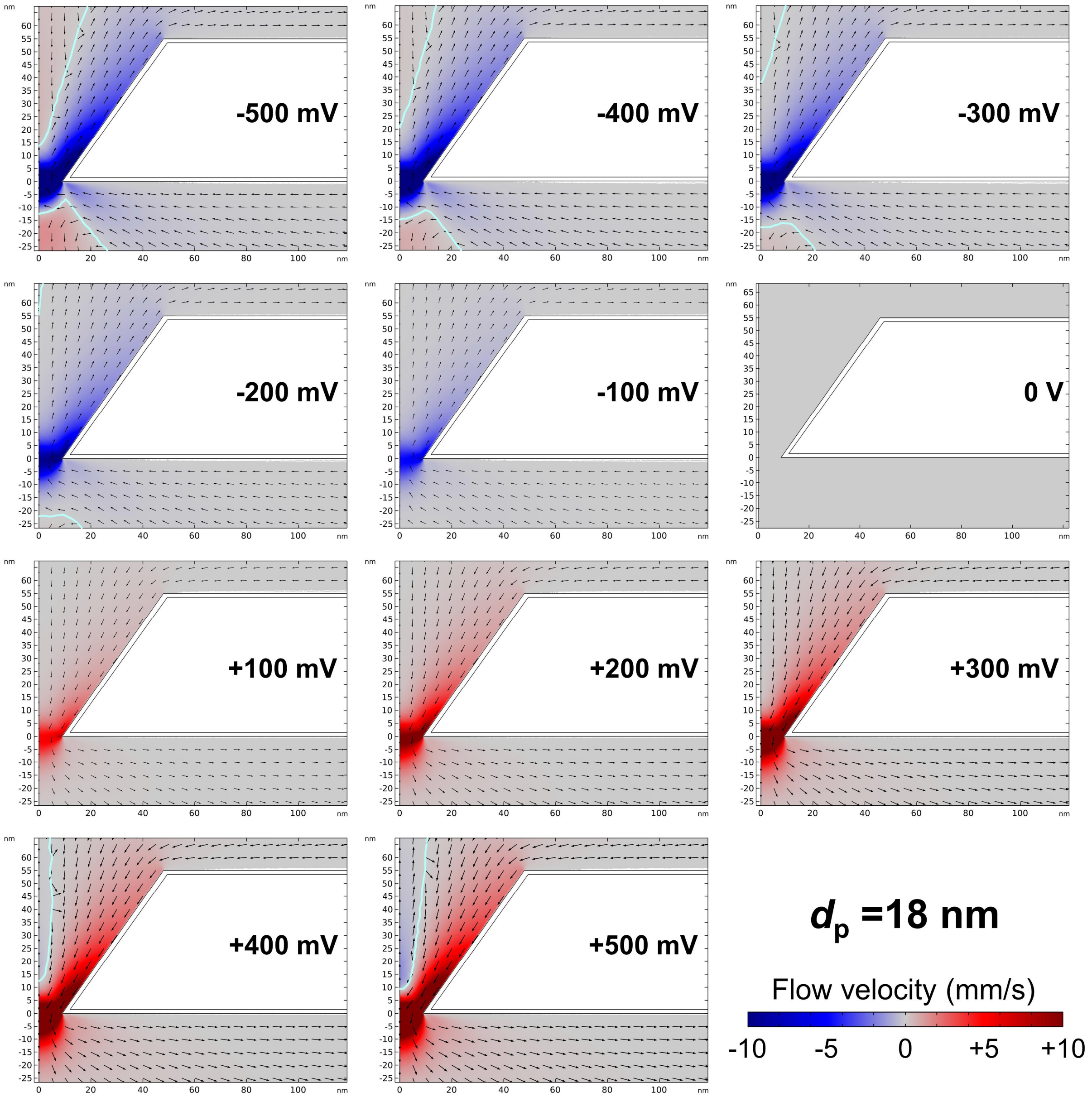

Figure S3. Distribution of EOF velocity for a TCP of d_p = 18 nm in the range of applied bias voltage from -500 mV to +500 mV. The cyan curves indicate the boundaries of the vortices.

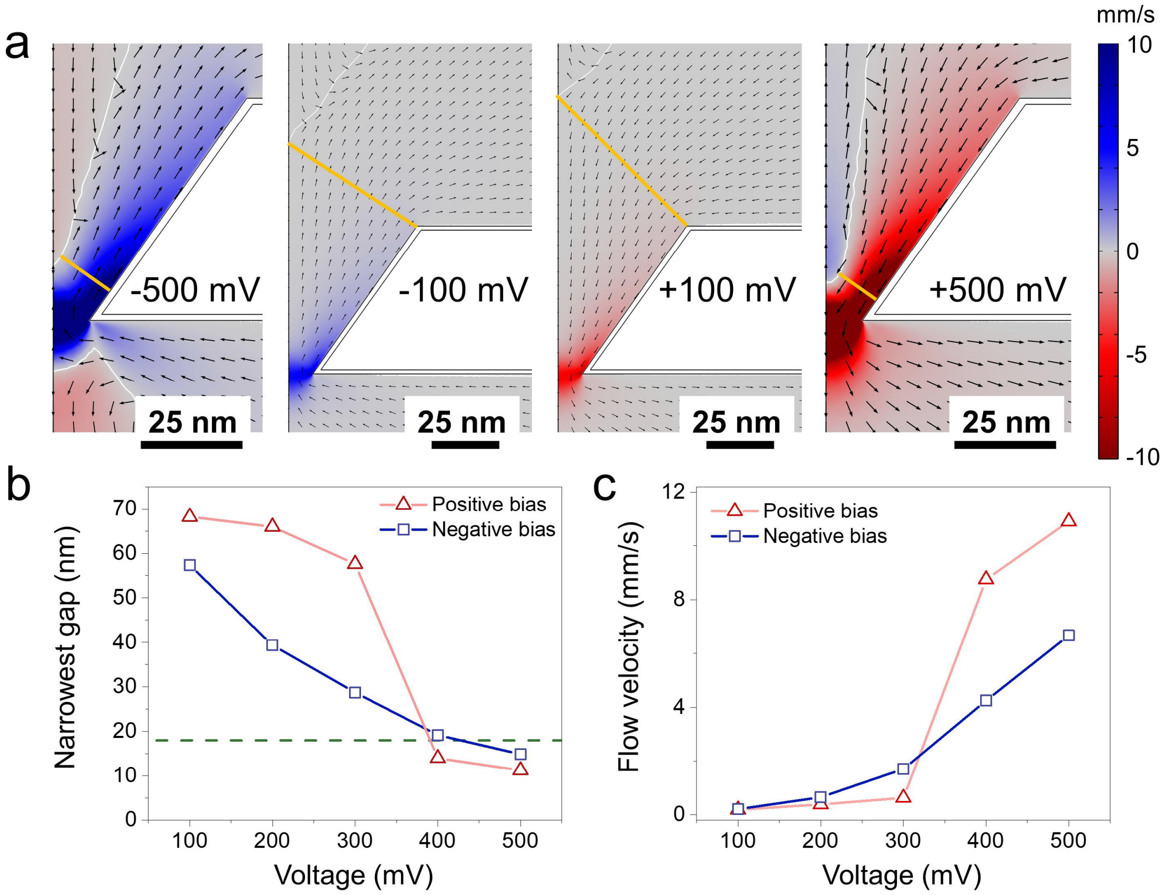

Figure S4. (a) Enlarged views of EOF velocity distribution for the case of d_p = 18 nm TCP biased at -500, -100, +100, and +500 mV. Yellow straight lines refer to the location of the narrowest gap between the nanopore sidewall and the vortex. Variation of (b) the narrowest gap measured by the yellow lines in (a) and (c) average EOF velocity with bias voltage from -500 mV to +500 mV for the case of d_p = 18 nm TCP.

Note 2. Particle-wall interaction in COMSOL simulation

The Particle Tracing Module in COMSOL Multiphysics provides a feature that the path of individual particles in fluids can be obtained by solving their equations of motion over time. When a particle encounters a wall, the following options are supported by the COMSOL code: disappear, freeze, stick, path through, bounce, diffuse scattering, isotropic scattering, mixed diffuse and specular reflection, and general reflection, respectively. During the simulation, the injected particles must be continuously present inside the fluid, so we could adopt the 'bounce' and 'diffuse scattering' options for our model ('diffuse scattering' used for interface between nanopore/water, while 'bounce' used for all other boundaries except for nanopore itself).

According to the COMSOL user guide,[10] the 'bounce' option is typically used when tracing microscopic particles in a fluid. Since the reflected angle is the same as the incident angle with respect to the surface normal, the velocity of particle after contact is given by

$$v_p = v - 2(\boldsymbol{n} \cdot v)\boldsymbol{n}, \quad \text{(S2)}$$

where v_{p} is the post-contact velocity, v is the pre-contact velocity, and $\boldsymbol{n}$ is the normal vector. On the other hand, the injected particles bounce off a wall according to the Knudsen's cosine law if we would choose the 'diffuse scattering' option. Thus, the reflected direction of particles has been determined by a given direction considering the probability within a solid angle $d\omega$ is given by $\cos(\theta)d\omega$, where θ is the angle between the direction of the reflected particle and the wall normal. For all preset options, the total particle kinetic energy is conserved.

If we want to make an inelastic boundary condition based on the 'bounce' option, which is available to modify Equation (S1) through the 'general reflection' option as follows

$$v_p = v - \alpha \cdot 2(\boldsymbol{n} \cdot v)\boldsymbol{n}, \quad \text{(S3)}$$

where α is a user-defined damping factor. If we set $\alpha = 0.9$, the particle will leave the wall with 90% of the incoming velocity. Note that α can also be a function of the velocity. Moreover, we also can set up a variety of particle-wall interactions through the 'general reflection' option.

Note 3. Brownian force equation in COMSOL simulation

From the Langevin equation, the equation of motion of particles only considering the Brownian force can be described as follows[11-14]

$$m\ddot{r} = -\gamma\dot{r} + F(r) + F_B, \tag{S4}$$

$$F_B = \sigma\xi(t), \tag{S5}$$

where m is the mass of particle (kg), r displacement (m), $\dot{r} = \frac{dr}{dt}$velocity (m/s), $\ddot{r} = \frac{d^2r}{dt^2}$ acceleration (m/s^2), γ friction constant (N·s/m), F the conservative force (N), F_B Brownian force (N), which is a stochastic variable with the amplitude σ (N), and ξ a random fluctuating factor (dimensionless). For a spherical object of radius R in water, the friction constant can be described by the Stokes' law

$$F = 6\pi\eta vR = \gamma v, \tag{S6}$$

$$\gamma = 6\pi\eta R. \tag{S7}$$

In the zero-mass limit, *i.e.*, the mass is so small and the friction force is so strong that the acceleration process happens with zero time.

$$\gamma\dot{r} = F(r) + \sigma\xi(t), \tag{S8}$$

$$\frac{dr}{dt} = \frac{F(r)}{\gamma} + \frac{\sigma}{\gamma}\xi(t). \tag{S9}$$

According to the fluctuation-dissipation theorem, the thermal fluctuation is motivated from its thermal energy 1.5kT and damped by the viscous media. It shows a white-noise-like Power Spectrum Density (PSD) that the powder density Γ is uniformly distributed on the frequency. (Indeed, the thermal noise is another typical example of fluctuation-dissipation mode in electronics system, so the PSD of a white thermal is $4kTR_{es}$ with R_{es} as resistance.)

$$PSD = \Gamma = 2\gamma kT. \tag{S10}$$

For a given bandwidth (BW) of the system, *i.e.*, the timestep, Δt, (sampling rate or time solution) of the observation in experiment or updating in simulation, the amplitude of the Brownian force is

$$F_B = \sigma = \sqrt{PSD \cdot BW} = \sqrt{\frac{2\gamma kT}{\Delta t}}. \tag{S11}$$

In water, it becomes

$$F_B = \sqrt{\frac{12\pi\eta RkT}{\Delta t}}, \tag{S12}$$

and is used in COMSOL simulation. Thus, the Langevin equation is expressed as

$$\frac{dr}{dt} = \frac{F(r)}{6\pi\eta R} + \sqrt{\frac{kT}{3\pi\eta R\Delta t}}\xi(t), \tag{S13}$$

$$\frac{dr}{dt} = F(r)\mu + \sqrt{2D\Delta t}\xi(t), \quad \text{(S14)}$$

$$\frac{dr}{dt} = u + \sqrt{2D\Delta t}\xi(t), \quad \text{(S15)}$$

where u is the drift velocity caused by force F as shown in this relation: $u = DF/kT = F\mu$ with μ as mobility. Here, μ in a wide sense is the ratio between the velocity and the applied force in a fully damped system (*i.e.*, an instantaneous acceleration). D is the diffusivity, from which the famous Stokes–Einstein relationship can be derived

$$D = \frac{kT}{6\pi\eta R} = \frac{\Gamma}{2\gamma^2} \quad \text{(S16)}$$

In our system, R ~ 3 nm; D ~ 8.4×10^{-11} m^2/s; m ~ 54.31 kDa = $54.31/6.02\times10^{23}$ = 9×10^{-23} kg;[15] T = 293.15 K.

A trade-off in the simulation is, as usual, between the timestep, Δt, and the duration to simulate for each of the large amount of translocation events, given the available computation resources. Choice of the former, Δt, needs to consider its direct influences on space step and F_B without yielding unphysical translocations. Our solution is to set a limit of at least two consecutive steps for an analyte to move and cross the central region of a TCP defined by L_{eff}. With this constraint, Δt = 0.25 μs is satisfactory because it also allowed for simulation of the translocation events each reaching 10 ms with numerous repetitions to minimize statistical errors.

Table S3. Brownian motion of spheres with similar physical properties of streptavidin (see main text or Note 4 of Supporting Information) in water with respect to the set timestep.

Timestep, Δt (μs)	0.05	0.10	0.25	0.50
F_B (pN)	2.79	1.97	1.25	0.88
Space step = $\sqrt{4Dt}$ (nm)	4.1	5.8	9.2	12.9
Velocity = $\sqrt{4D/t}$ (m/s)	0.082	0.058	0.037	0.026
Root-mean-square velocity = $\sqrt{3k_BT/m}$	11.6 m/s			

Note 4. Random vector generator for evaluating the Brownian force

Based on the Langevin equation, the Particle Tracing Module in COMSOL Multiphysics basically uses the governing equation of the F_B that can be expressed as[10]

$$F_B = \zeta\sqrt{\frac{6\pi\eta k_B T d_a}{\Delta t}}, \quad \text{(S17)}$$

where d_a, ζ, k_B, η, T, and Δt are the diameter of the sphere (m), generated random vector (dimensionless), Boltzmann constant (J/K), viscosity (Pa·s), absolute temperature of the fluid (K), and timestep (s) taken by the solver, respectively.[11, 12] ζ turns out to be a Gaussian distributed pseudorandom number with zero-mean and unit variance,[11, 16] which allows for a good representation of the Brownian motion for injected particles in the simulation. For instance, Li and Ahmadi reported that the simulation results were in good agreement with the theoretical prediction when comparing the time-dependent root-mean-square (RMS) displacement for 500 massless particles.[11] This result implies that Equation (S16) would perform well in the simulation from a statistical perspective, but it needs to be handled carefully when trying to observe the trajectory of very small individual particles of ~nm in dimension due to the Gaussian distribution in ζ by the default built-in 'randomnormal' function.

Note that a unit variance does not mean that it cannot exceed 1; there is in fact 68% chance that it is lower than 1, but still probable that it is larger than 1. Thus, we compared the results of changing the random vector generator (Figure S5 versus S6), and confirmed that the 'random' function was necessary to build the model to observe the trajectory of individual particles in our nanopore system. In these two simple simulations, 20 particles all assuming the density (ρ = 820 kg/m^3)[15] and diameter (d = 6 nm)[5, 17] of streptavidin were released into the 2D square-shaped water (100 nm × 100 nm) at room temperature. The only difference is that ζ was defined as 'randomnormal' (Figure S5) or 'random' (Figure S6) when obtaining the Brownian motion of particles in the time range of 0 – 100 μs. Contrary to the 'randomnormal' function, the built-in 'random' function makes a uniformly distributed pseudorandom number between -0.5 and +0.5, which does not exceed 1. This result implies that while the Brownian motion can be well described using the 'randomnormal' function, there may be cases with which an overestimated random number near the nanopore orifice dominates the other forces, which would not be the case in the real world. On the other hand, with the 'random' function, we could get a similar result by applying a low-pass filter on the magnitude while maintaining the random behavior as when using the 'randomnormal' function. All this can be seen more clearly by comparing the RMS values of F_B in each case to the results in Table S3.

Therefore, we used two different random vector generators (‘randomnormal’ and ‘random’) in our simulations. Near the nanopore orifice, the ‘random’ function was found to be more appropriate to prevent an overestimated F_B from leading to unphysical transport behaviors of particles. On the other hand, the ‘randomnormal’ function was more suitable in the rest of the system because it could allow the particles to move near the nanopore orifice more frequently, which could account for the experimentally observed frequent translocations.[6] Moreover, according to the user guide of Particle Tracing Module, the ‘randomnormal’ option is used sometimes instead of the ‘random’ option, which could support that our use of the ‘random’ option in some areas of our simulation from a systemic perspective.

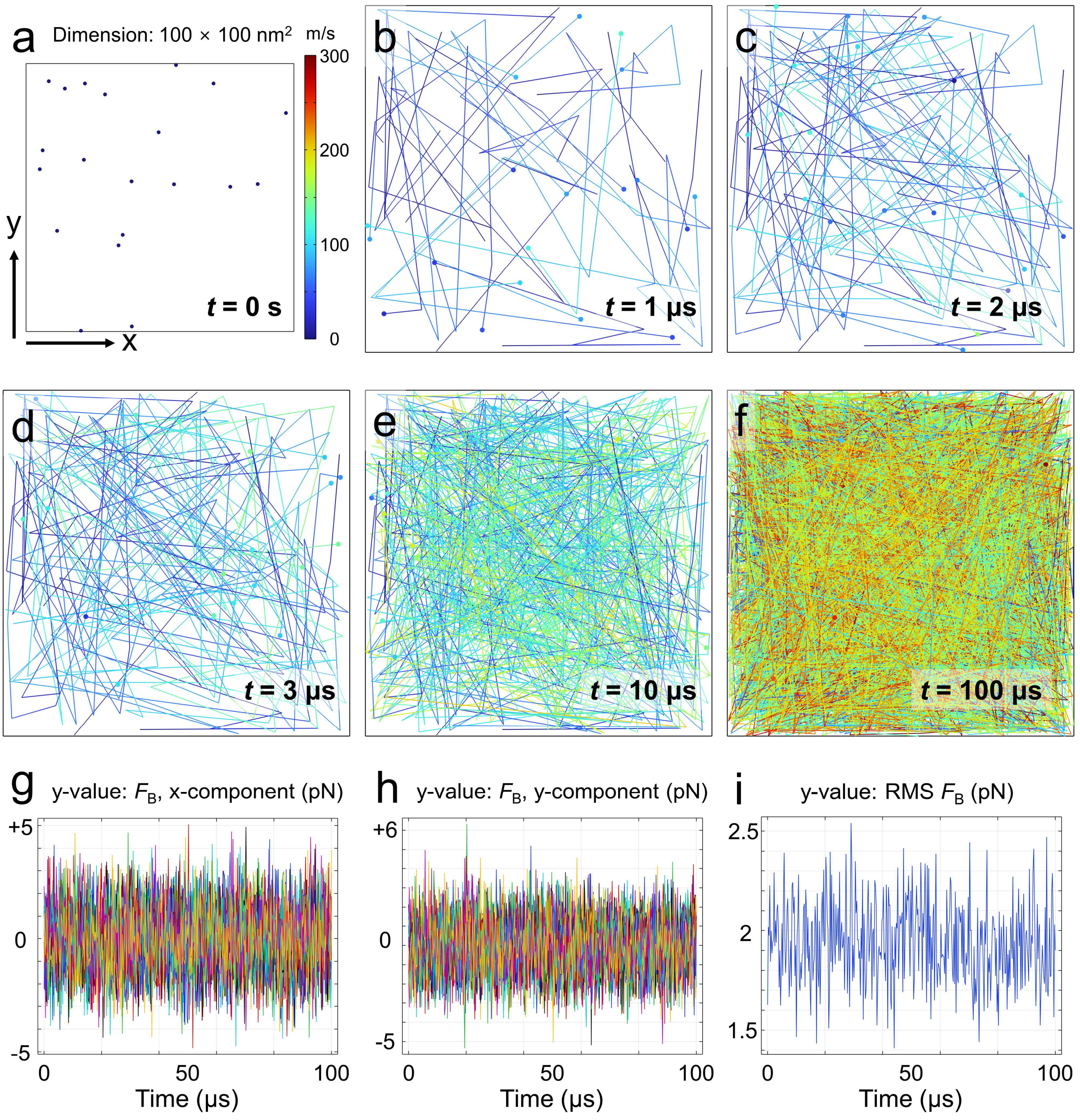

Figure S5. Tracking 20 analytes (with the properties of streptavidin, see main text) over time, *t*, in the range of 0 to 100 μs with a 0.25 μs timestep and a 'randomnormal' function. (a) *t* = 0 μs, (b) *t* = 1 μs, (c) *t* = 2 μs, (d) *t* = 3 μs, (e) *t* = 10 μs, (f) *t* = 100 μs, with reference to the coordinates of the 2D rectangular water (dimension: 100 × 100 nm^2) in (a). Time dependence of F_B of individual analytes along (g) x-axis and (h) y-axis in (a). (i) Variation of the root-mean square (RMS) F_B on a typical analyte with time for the 20 analytes.

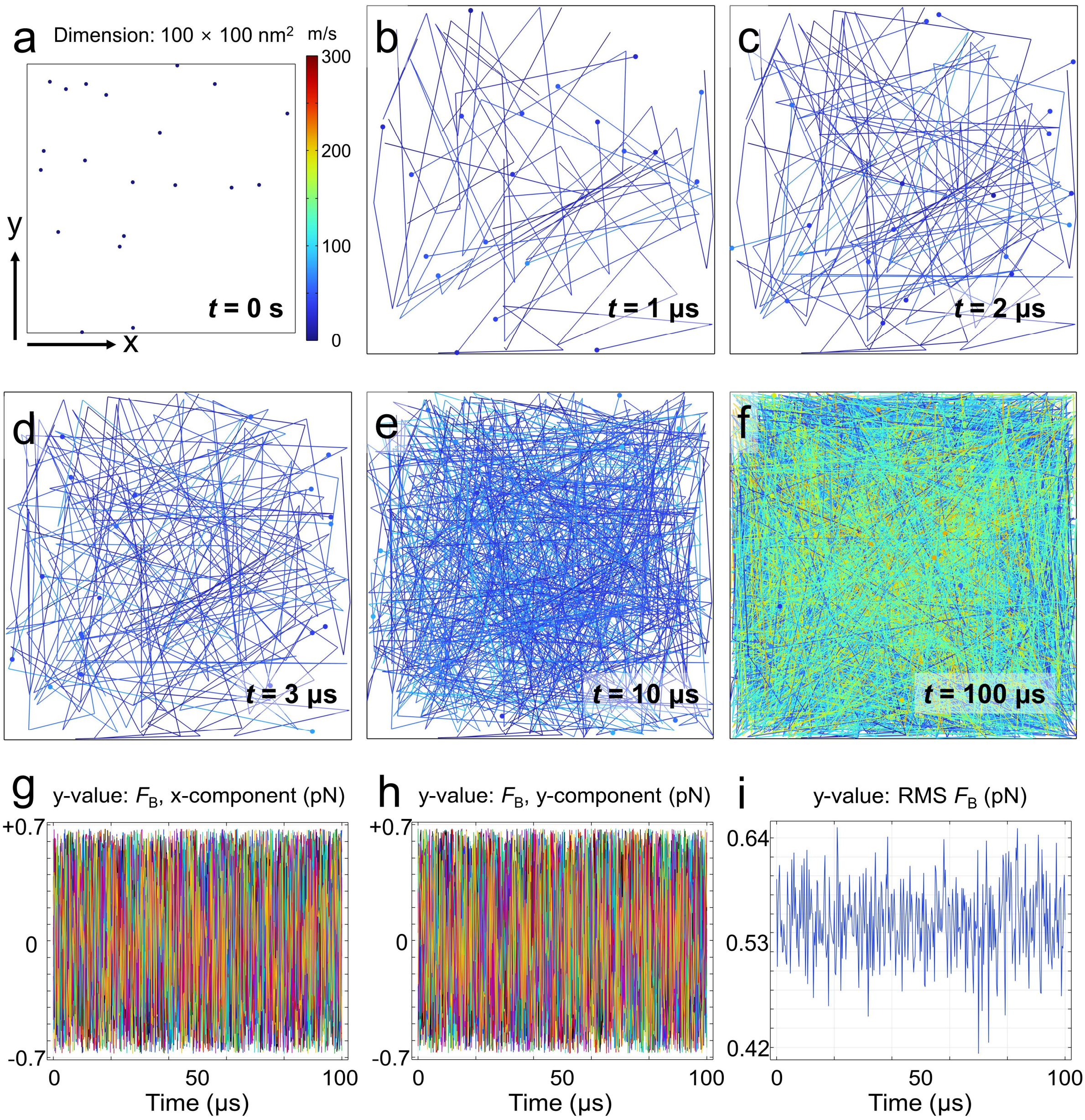

Figure S6. Tracking 20 analytes (with the properties of streptavidin, see main text) over time, t, in the range of 0 to 100 μs with a 0.25 μs timestep and a 'random' function. (a) t = 0 μs, (b) t = 1 μs, (c) t = 2 μs, (d) t = 3 μs, (e) t = 10 μs, (f) t = 100 μs, again with reference to the coordinates of the 2D rectangular water (dimension: 100 × 100 nm^2) in (a). Time dependence of F_B of individual analytes along (g) x-axis and (h) y-axis in (a). (i) Variation of the root-mean square (RMS) F_B on a typical analyte with time for the 20 analytes.

Note 5. Definition of translocation in COMSOL simulation

When setting up the experimental conditions, the experimental concentration[5] of streptavidin (*i.e.*, analytes) at 84 nM in each reservoir is considered well maintained regardless of the measurement time because the huge number of continually injected streptavidin is incomparably large compared to the translocated, "lost" analytes. Contrary to experiment, however, there is possibility that the concentration of analytes in each reservoir differs from the experimental value due to the continuous translocations since a finite number of 319 analytes is introduced in each reservoir in the simulation. At $t = 0$ s, all analytes are released in the reservoirs (region 1 and 2 in Figure S7) based on the 'density' option in the Particle Tracing Module. The initial position of the analytes looks a bit random, but the distribution depends on the underlying mesh. Over time, all injected analytes are predominantly driven by Brownian motion and move randomly in the zones marked in Figure S7. Once they wander into the high electric field area in the vicinity of nanopore, characterized by effective transfer length, L_{eff}, the strong electroosmotic force (EOF) can push them through the pore. For those few analytes that eventually travel along and complete the path 'zone 1 → 2 → 3 → 4' or 'zone 4 → 3 → 2 → 1', one translocation is registered in the simulation. To ensure that the concentration in both reservoirs is consistent, the analytes that have completed their passage are immediately removed and new analytes are subsequently reintroduced into (or cloned in) their respective original zones realized by employing the built-in 'Velocity Reinitialization' feature of COMSOL Multiphysics.

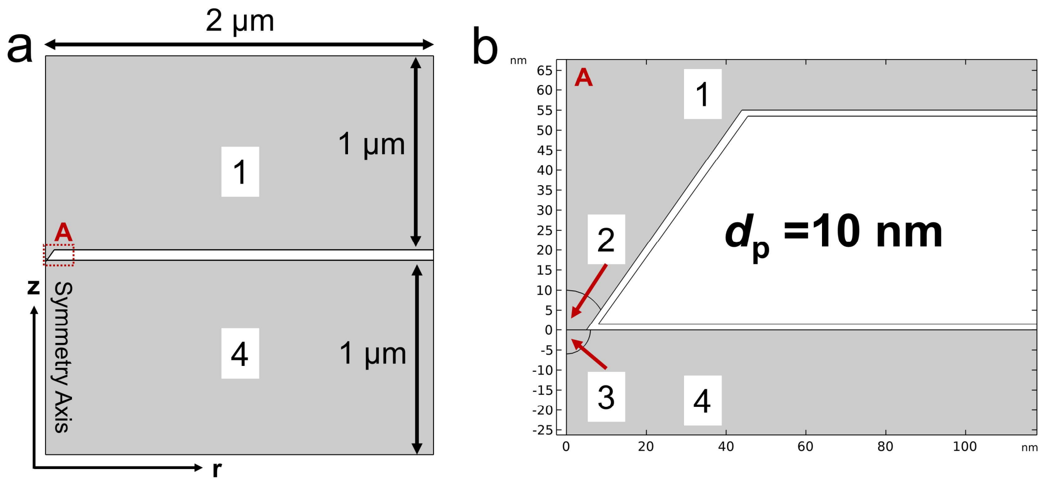

Figure S7. Zones 1 – 4 defined in the model for registering translocation events. (a) Zones 1 and 4 wherein analytes are initially placed. (b) Zones 2 and 3 also displayed for the case of d_p = 10 nm. Black curves immediately above and below the nanopore orifice mark where L_{eff} extends to in zones 2 and 3.

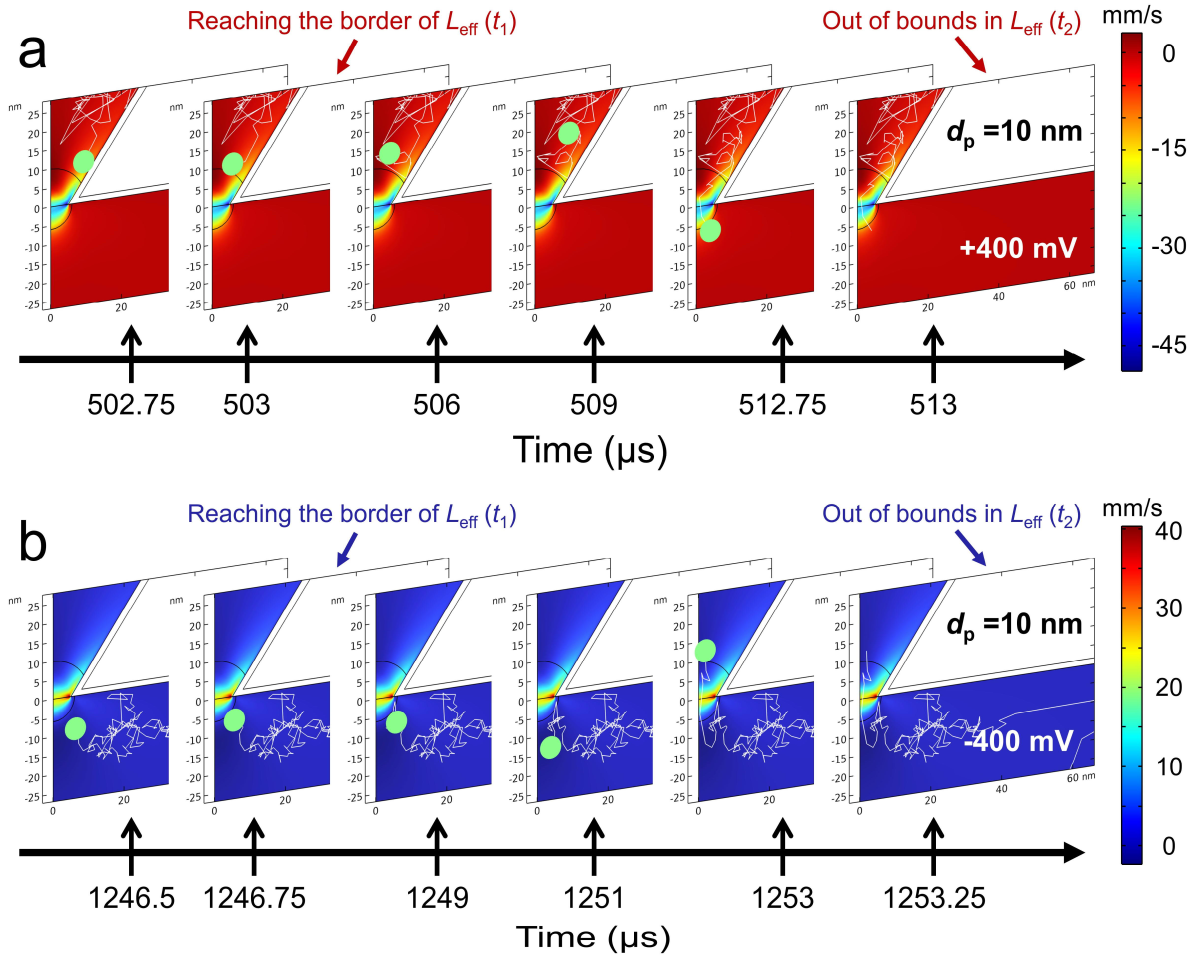

Figure S8. Determination of the dwell time ($t_2 - t_1$) of successful translocations in COMSOL simulation. (a) d_p = 10 nm, +400 mV and (b) d_p = 10 nm, -400 mV. Green circles: analytes being tracked; connected short white lines: trajectory of the analytes.

Note 6. Properties of streptavidin used in COMSOL simulation

It has been reported that streptavidin (3RY1) has a sphere-like shape with a diameter of 6 nm with a total volume of 110 nm^3.[17] Since its molecular weight is given as 54.31 kDa in Protein Data Bank,[15] the mass density is estimated to ~820 kg/m^3 in the simulation. Furthermore, the properties regarding the isoelectric point and net charge of proteins are obtained from an online protein calculator (https://www.protpi.ch/Calculator/ProteinTool). For streptavidin, it is a tetramer (4-mer) so that all the sequences from Protein Data Bank are copied to obtain its net charge. Finally, the isoelectric point and number of net charge (Z) at pH 7.4 are determined to be 6.16 and -5.346, respectively, as shown in Figure S9. Consideration of the effect of salt concentration, in addition to pH, on Z results in an effective net charge used in the simulation (see main text). In general, the effective net charge value decreases with increasing salt concentration.[18, 19]

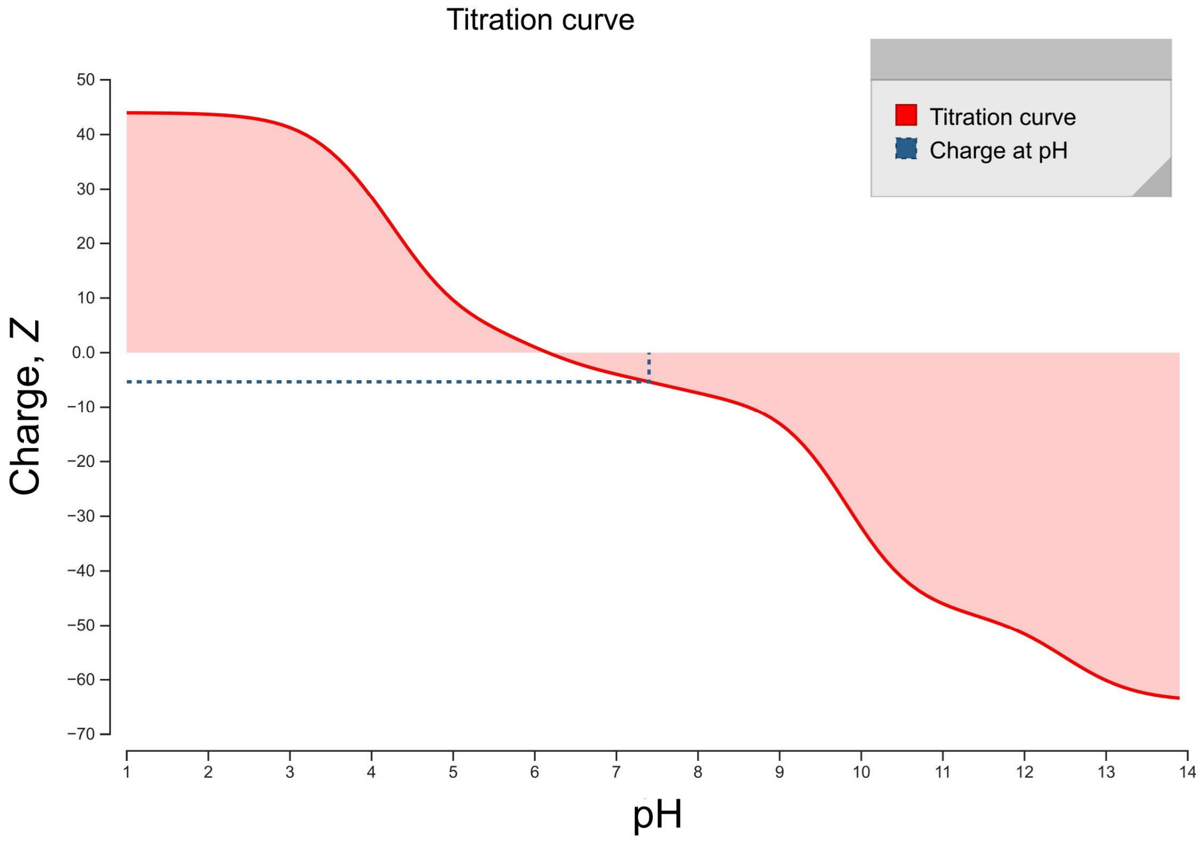

Figure S9. Calculated titration curve of streptavidin (3RY1) as a function of pH value.

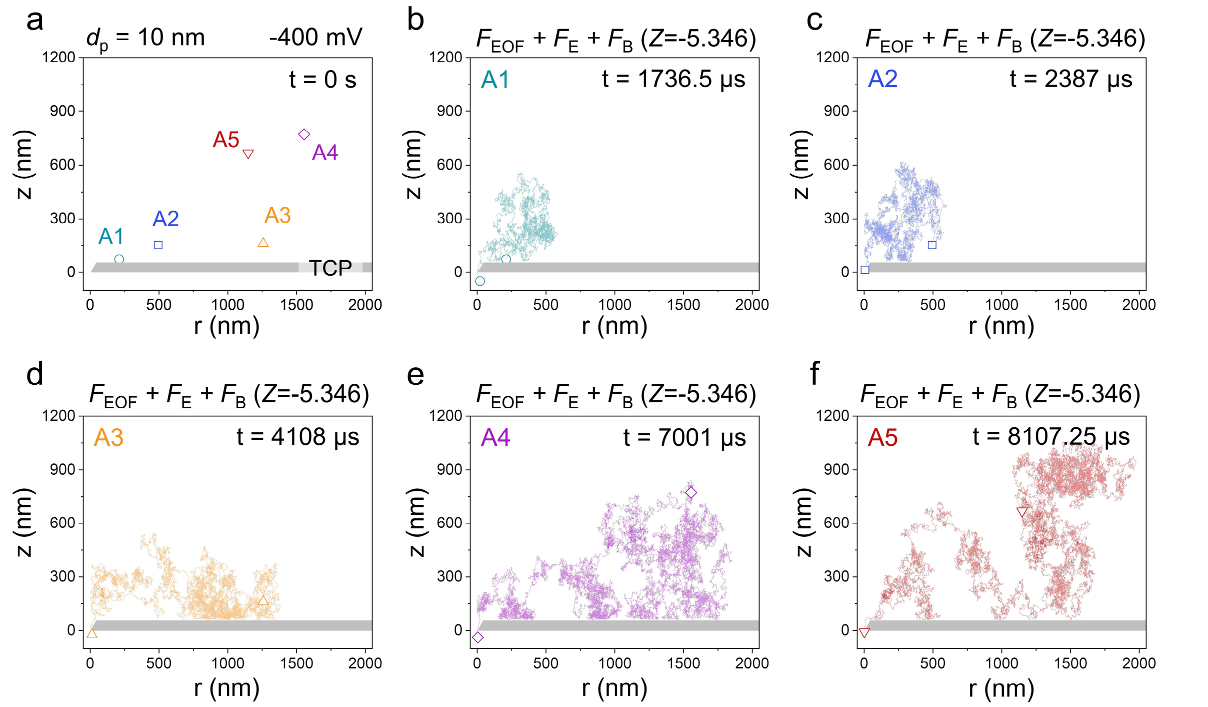

Figure S10. Trajectory plots of five randomly selected analytes (A1 – A5, Z = -5.346) at different time points t: (a) 0 (A1 – A5), (b) 1736.5 (A1), (c) 2387 (A2), (d) 4108 (A3), (e) 7001 (A4), and (f) 8107.25 μs (A5) for d_p = 10 nm by considering F_{EOF}, F_E, and F_B under the bias voltage of -400 mV.

Note 7. Effective net charge used in COMSOL simulation

The net charge of streptavidin is known to be affected by not only the pH value of an electrolyte (as discussed in Note 5) but also the salt concentration in the electrolyte.[18, 19] Thus, the effect of net charge on the translocation of streptavidin is investigated in Figure S11–S13. First, the Brownian force F_B was left out and only drag force F_D and electric force F_E were considered in Figure S11 and S13a. In Figure S11, the vicinity around the nanopore orifice is distinct with a severe depletion of analytes due to Coulomb repulsion imposed by F_E (Figure S13a) along with the assumption of no F_B to diffuse analytes to the depleted region. Furthermore, the direction of translocation dominated by F_E is opposite to that observed in our experiment.[5] In our second setup, the effective Z of analytes was applied instead of its pristine Z at pH 7.4 (Note 6) and F_B was included. In Figure S12, the translocation behaviors of analytes at different effective Z and with F_B included are displayed. No depletion is seen as analytes from outside the L_{eff} region can now be delivered into the pore region by F_B. More importantly, the direction of analyte translocation consistently shifts from being governed by F_{EOF} to that by F_E when the magnitude of Z is changed from -0.1, to -1, and to -5.346 (Figure S13b). The trend with Z = -0.1 is satisfactorily in good agreement with the experimental observations.[5] Therefore, the simulation data presented in the main text were conducted with the assumption of Z = 0 for the analytes rather than finding an exact Z value in the highly salt concentrated 5× PBS in order to conform to the F_{EOF}-dominated translocation.

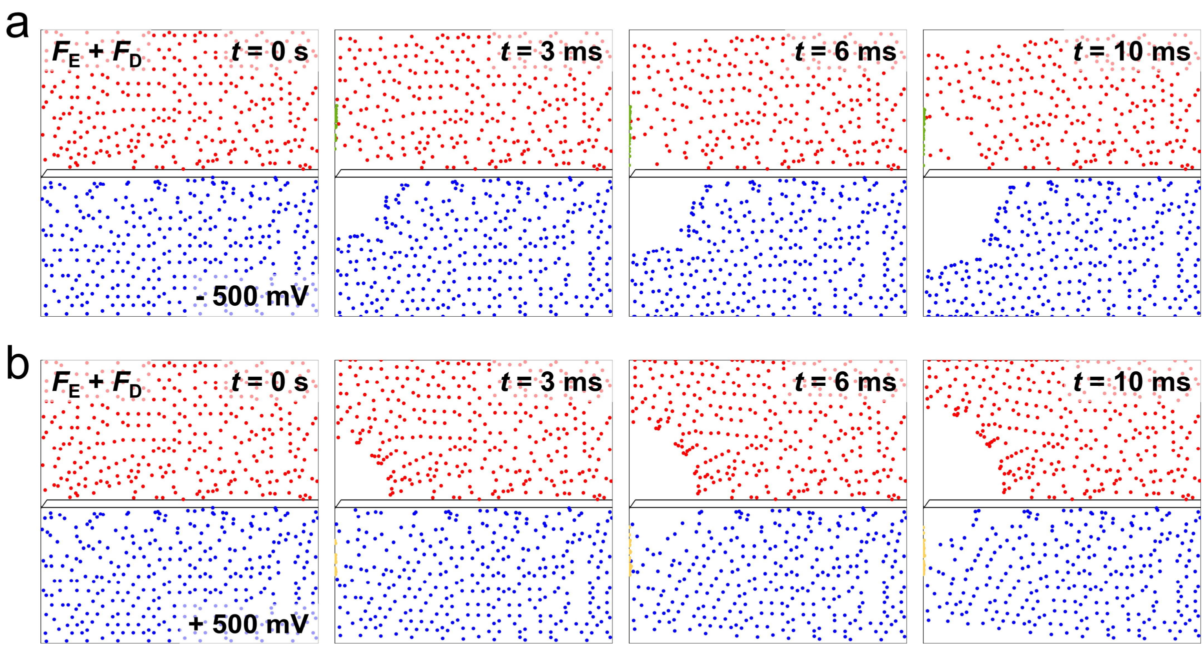

Figure S11. Time-dependent analyte distribution (Z = -5.346) at different time point t = 0, 3, 6, and 10 ms) for d_p = 10 nm. (a) Negative (-500 mV) and (b) positive (+500 mV) bias voltages, when only considering drag (F_D) and electric or electrophoretic (F_E) forces. Red and blue circular dots refer to the primary analytes placed in the upper and lower reservoirs at t = 0 s. Green and yellow circular dots represent secondary analytes created in the upper and lower reservoirs due to translocations over time in order to retain the analyte concentrations invariant; each green is generated upon a "lost" red whereas each yellow is for a "lost" blue. All analytes irrespective of the color of the dots have the same properties. Severe depletion of analytes in the vicinity of the nanopore orifice due to translocations to the opposite side of the nanopore is evident. The green dots in the upper reservoir and the yellow dots in the lower reservoir were, for simplicity, placed along the central symmetric axis.

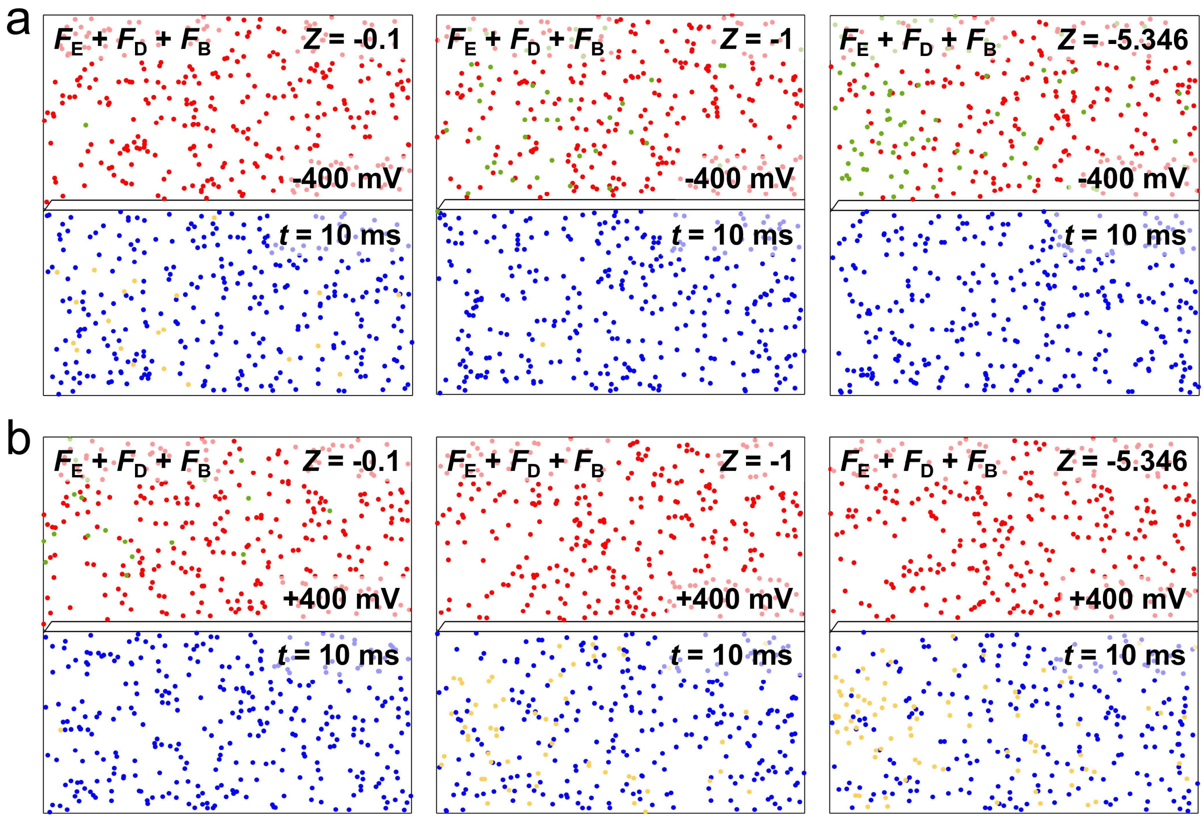

Figure S12. Analyte distribution at 10 ms for d_p = 10 nm with respect to the effective charge number of analytes, Z = -0.1, -1, and -5.436. (a) Negative (-400 mV) and (b) positive (+400 mV) bias voltages, by also considering the Brownian force F_B in addition to F_D and F_E. The analyte distribution remains random with neither obvious depletion of analytes in the vicinity of the nanopore orifice nor line-up of the translocated dots along the central symmetric axis. Moreover, the population of the green dots in the upper reservoir increases and that of yellow dots in the lower reservoir decreases with the absolute value of Z at -400 mV (and the opposite trends at +400 mV), thereby showing a distinct shift of the dominant force from F_{EOF} to F_E. The color code for the circular dots refers to the caption of Figure S11.

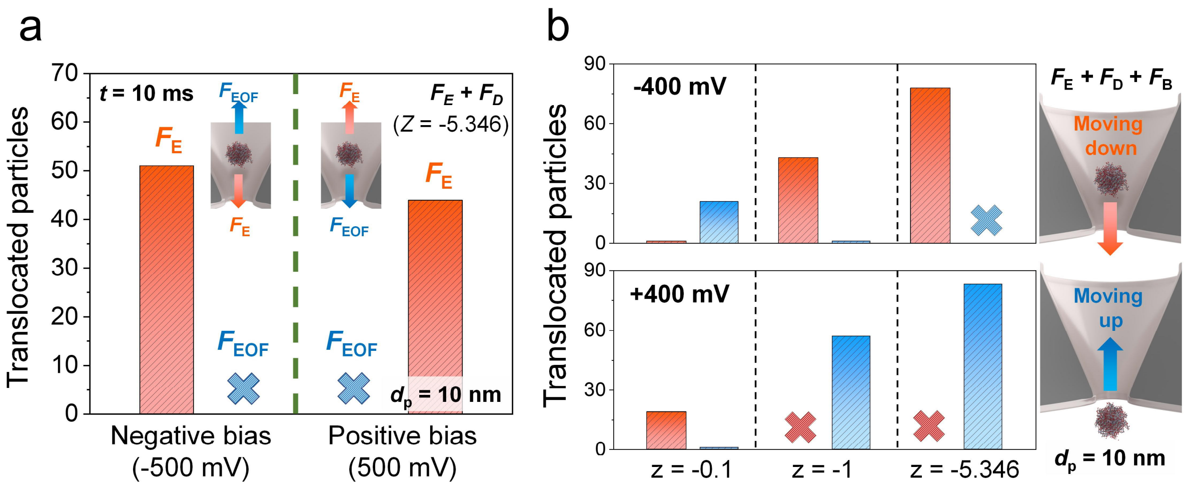

Figure S13. Summary of translocated analytes during 10 ms for the d_p = 10 nm nanopore as shown in Figure S11 and S12. (a) The translocation of negatively charged analytes (Z = -5.346) only in the presence of F_E and F_D under bias voltages of ±500 mV. (b) Comparison of Z-dependent translocation for Z = -0.1, -1, and -5.346, by considering F_E, F_D, and F_B under bias voltages of ±400 mV.

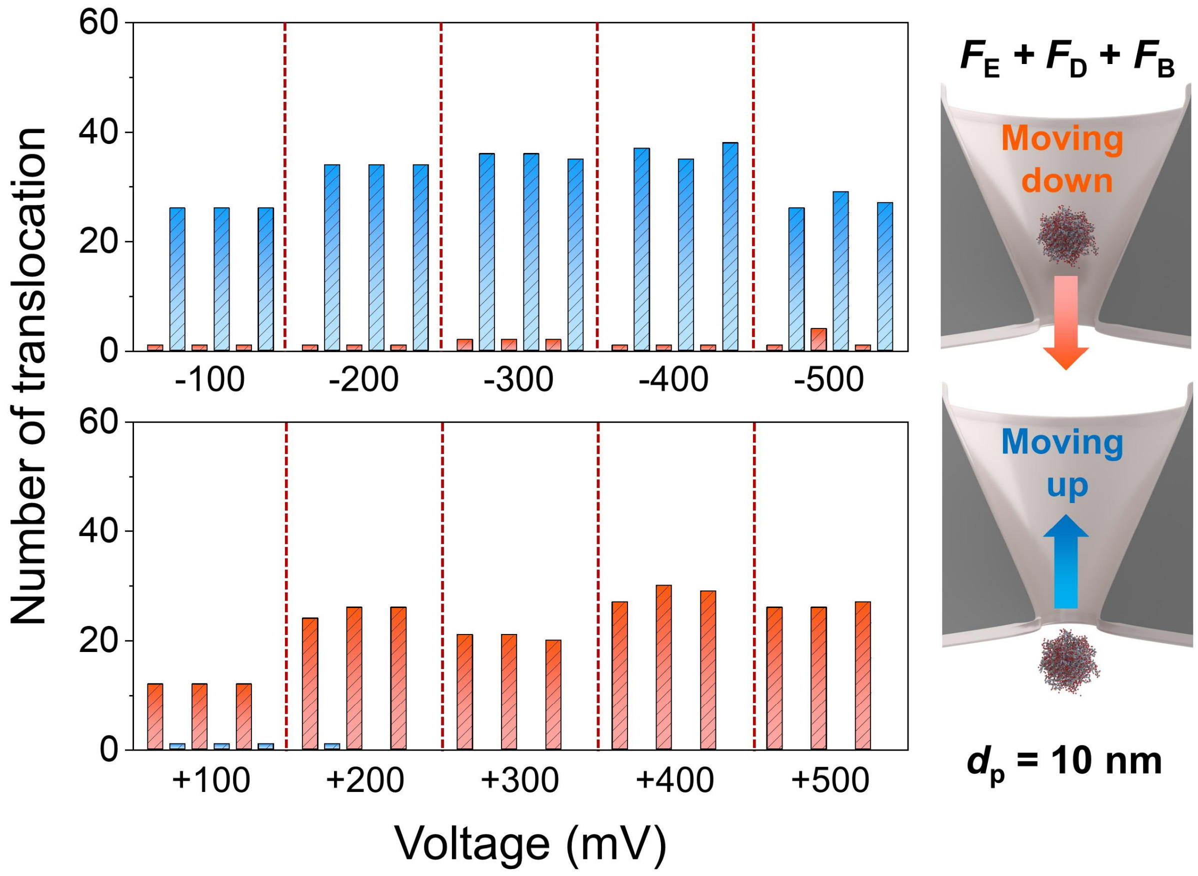

Figure S14. Summary of translocated analytes for d_p = 10 nm as a function of bias voltages. All the three forces (F_D, F_E, and F_B) were considered during the translocation. Red and blue color represent the translocation direction by F_{EOF} under positive and negative bias voltage. Due to the randomness caused by F_B, the simulations were repeated three times at each bias voltage.

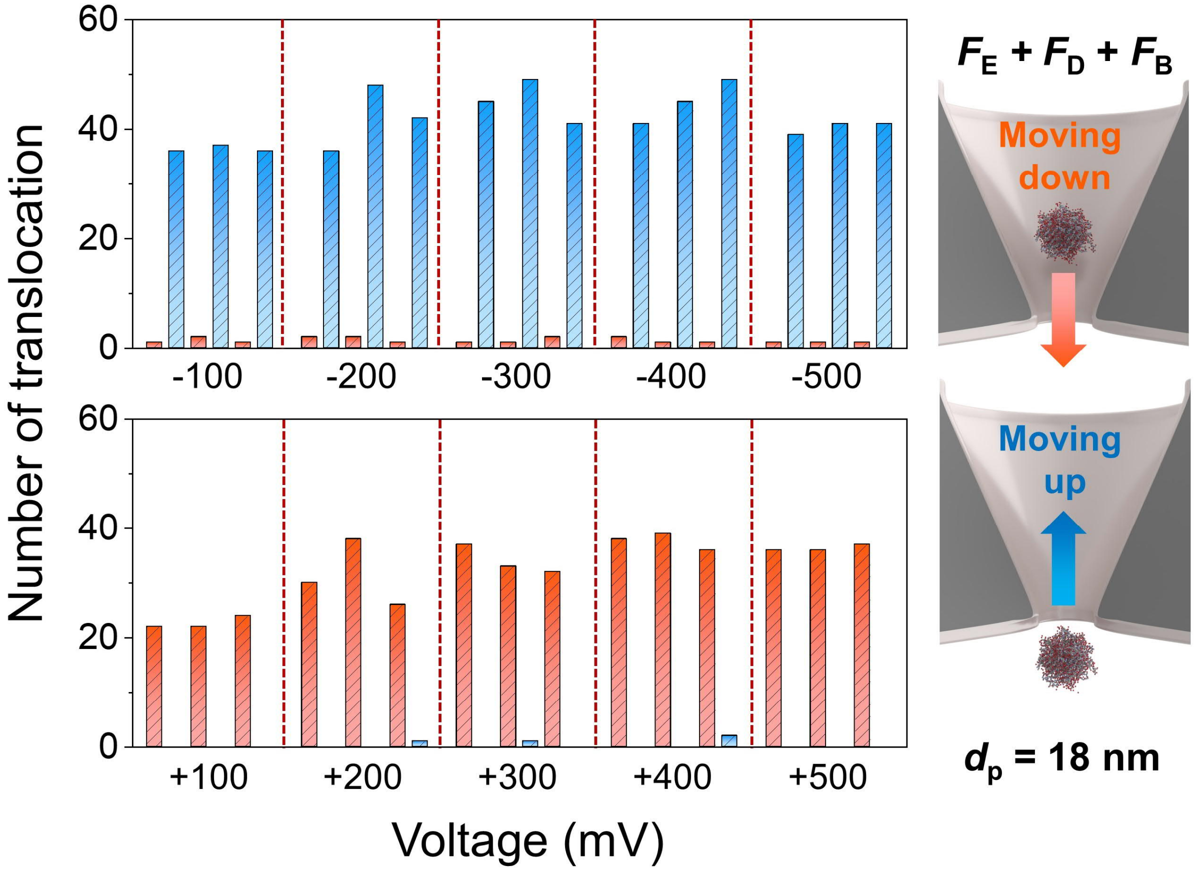

Figure S15. Summary of translocated analytes for d_p = 18 nm as a function of bias voltages. Due to the randomness caused by F_B, the simulations were repeated three times at each bias voltage. Description of applied forces and colors refers to the caption of Figure S14.

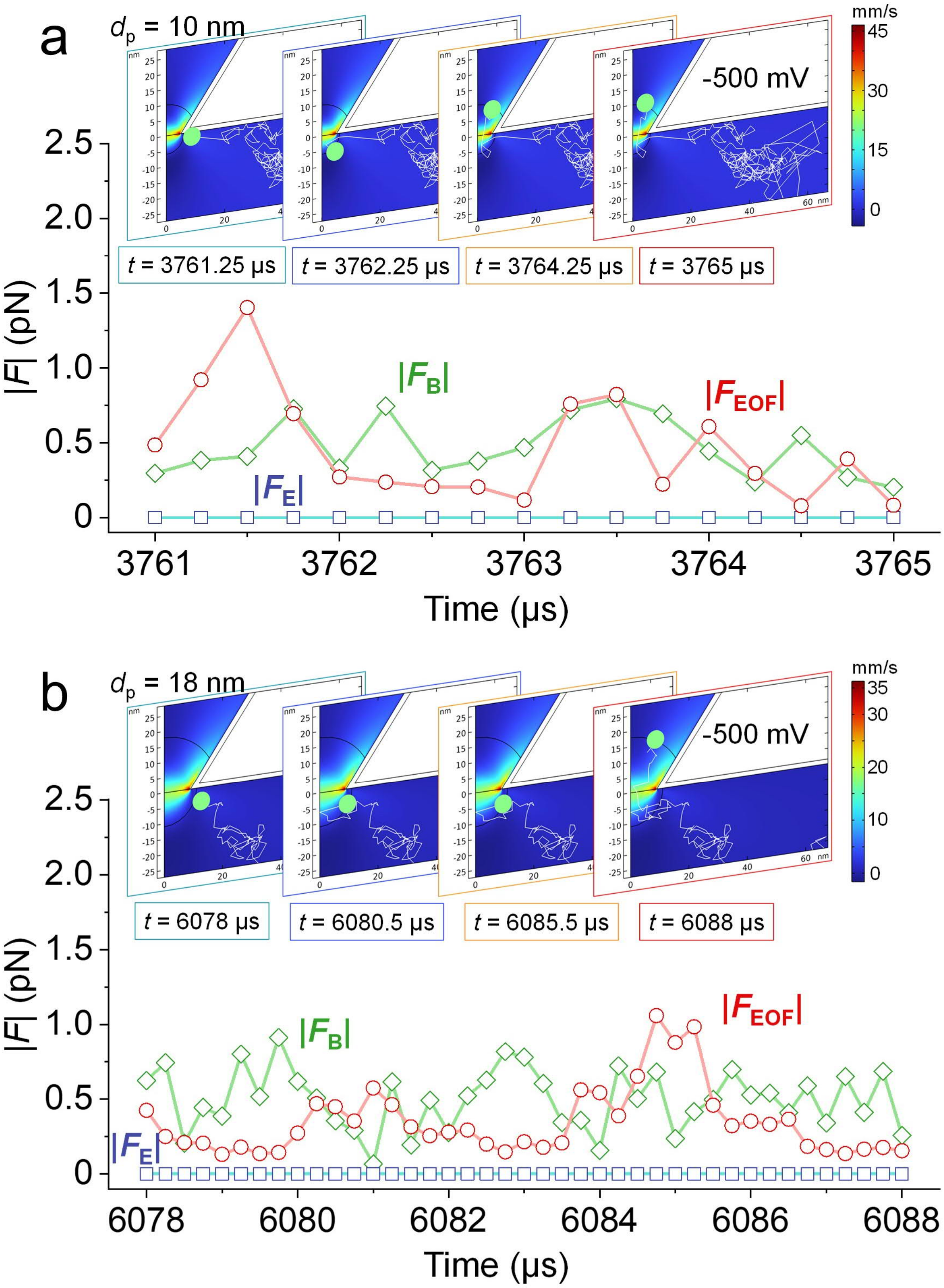

Figure S16. Time-dependent trajectory and corresponding forces of an analyte moving from the lower to the upper reservoir at -500 mV. (a) d_p = 10 nm and (b) d_p = 18 nm.

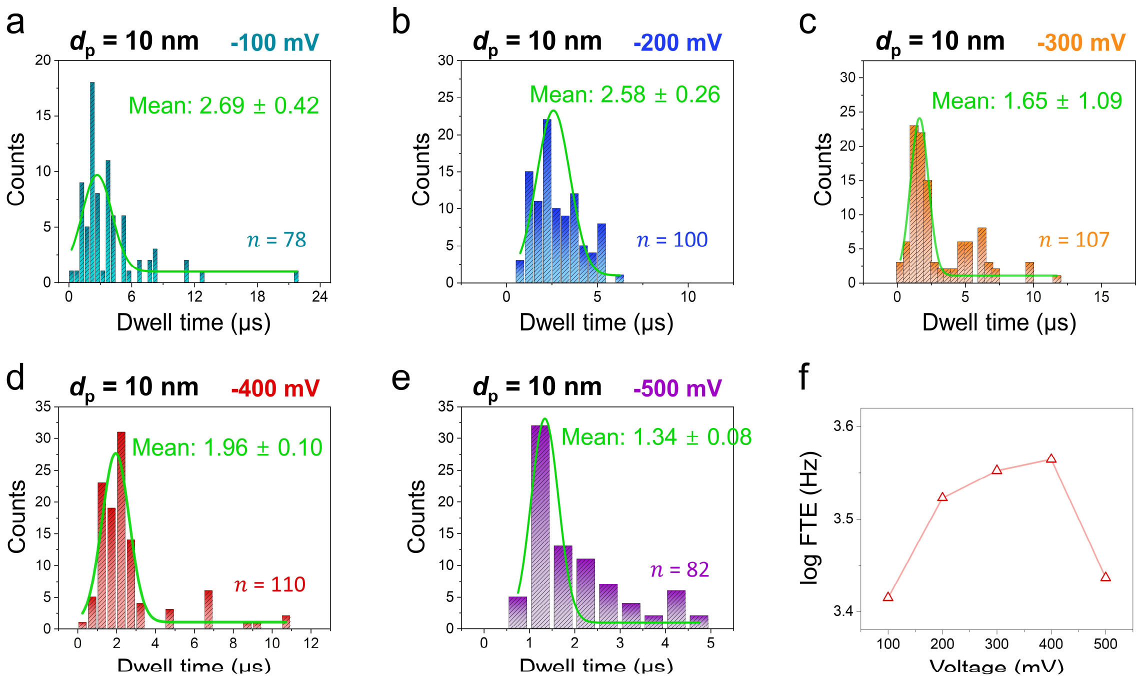

Figure S17. (a-e) Histograms of dwell time for d_p = 10 nm at different negative bias voltages ranging from -100 to -500 mV. The light green solid lines refer to the results of a Gaussian curve fitting with the baseline of 1. The number of counted analytes is denoted by '*n*'. (f) Logarithmic FTE in Hz versus voltage based on simulated FTEs during 10 ms.

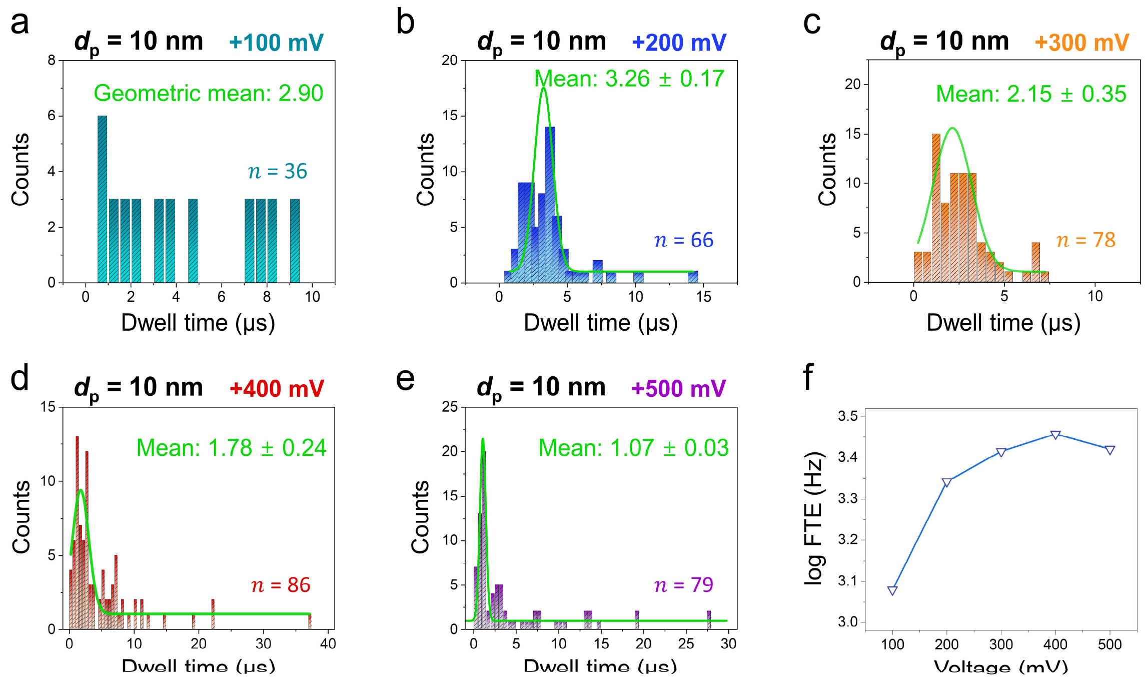

Figure S18. (a-e) Histograms of dwell time for d_p = 10 nm at different positive bias voltages ranging from +100 to +500 mV. The light green solid lines refer to the results of a Gaussian curve fitting with the baseline of 1. The number of counted analytes is denoted by '*n*'. (f) Logarithmic FTE in Hz versus voltage based on simulated FTEs during 10 ms.

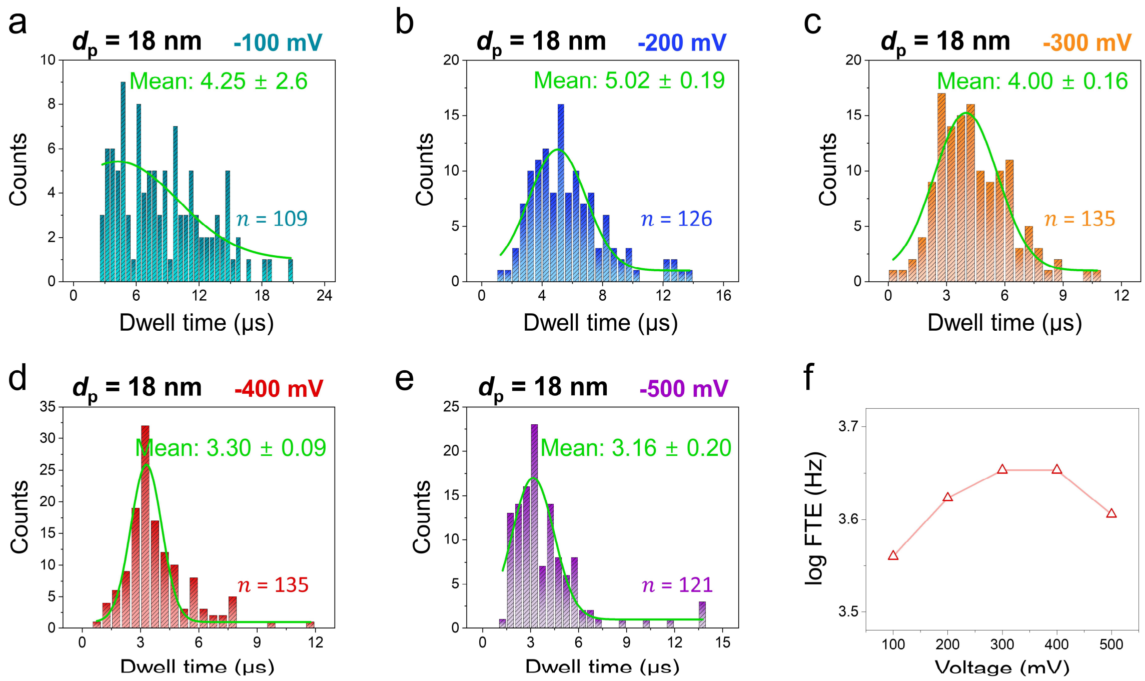

Figure S19. (a-e) Histograms of dwell time for d_p = 18 nm at different negative bias voltages ranging from -100 to -500 mV. The light green solid lines refer to the results of a Gaussian curve fitting with the baseline of 1. The number of counted analytes is denoted by '*n*'. (f) Logarithmic FTE in Hz versus voltage based on simulated FTEs during 10 ms.

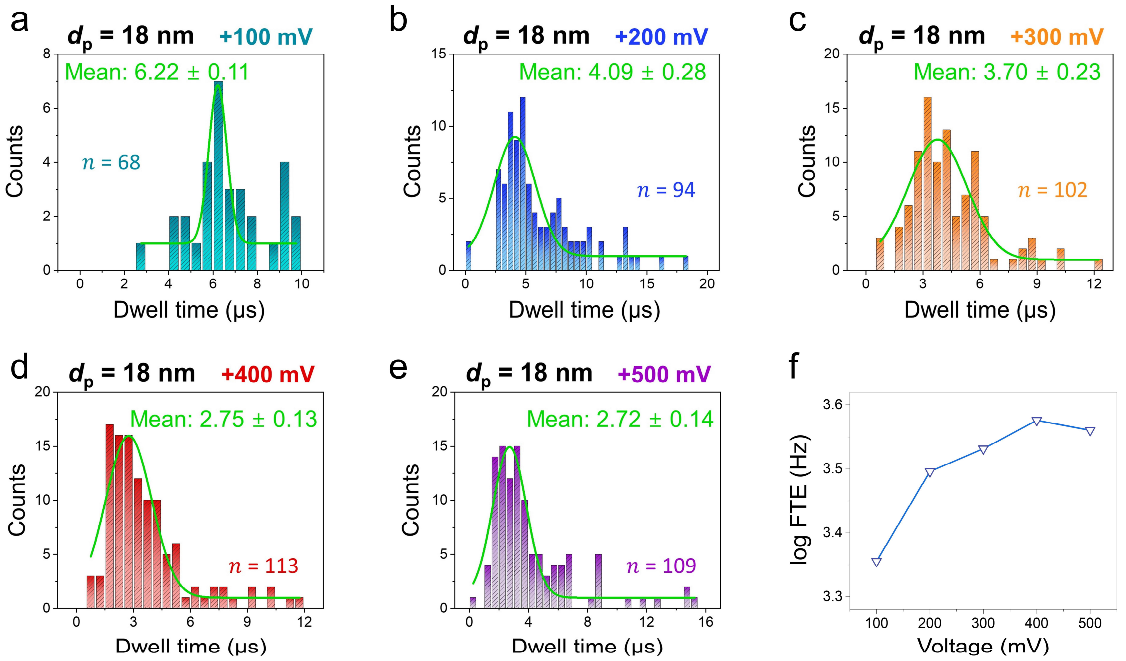

Figure S20. (a-e) Histograms of dwell time for d_p = 18 nm at different positive bias voltages ranging from -100 to -500 mV. The light green solid lines refer to the results of a Gaussian curve fitting with the baseline of 1. The number of counted analytes is denoted by '*n*'. (f) Logarithmic FTE in Hz versus voltage based on simulated FTEs during 10 ms.

References

1. Yao, Y.; Wen, C.; Pham, N. H.; Zhang, S.-L., *Langmuir* **2020,** *36*, 8874-8882.
2. Wen, C.; Zhang, S.-L., *Journal of Applied Physics* **2021,** *129*, 064702.
3. Samson, E.; Marchand, J.; Snyder, K. A., *Materials and Structures* **2003,** *36*, 156-165.
4. Table of Diffusion Coefficients. https://www.aqion.de/site/diffusion-coefficients.
5. Zeng, S.; Wen, C.; Solomon, P.; Zhang, S.-L.; Zhang, Z., *Nature Nanotechnology* **2019,** *14*, 1056-1062.
6. Wen, C.; Zhang, Z.; Zhang, S.-L., *ACS Sensors* **2017,** *2*, 1523-1530.
7. Feng, D.; Li, X.; Wang, X.; Li, J.; Zhang, T.; Sun, Z.; He, M.; Liu, Q.; Qin, J.; Han, S., *Chemical Engineering Science* **2018,** *186*, 228-239.
8. Fabuss, B. M.; Korosi, A.; Othmer, D. F., *Journal of Chemical & Engineering Data* **1969,** *14*, 192-197.
9. Othmer, D. F.; Conwell, J. W., *Industrial & Engineering Chemistry* **1945,** *37*, 1112-1115.
10. *Particle Tracing Module User's Guide, COMSOL Multiphysics® v. 6.1*. COMSOL AB, Stockholm, Sweden: **2022**.
11. Li, A.; Ahmadi, G., *Aerosol Science and Technology* **1992,** *16*, 209-226.
12. Li, T.; Raizen, M. G., *Annalen der Physik* **2013,** *525*, 281-295.
13. Huang, R.; Chavez, I.; Taute, K. M.; Lukić, B.; Jeney, S.; Raizen, M. G.; Florin, E.-L., *Nature Physics* **2011,** *7*, 576-580.
14. Li, T.; Kheifets, S.; Medellin, D.; Raizen, M. G., *Science* **2010,** *328*, 1673-1675.
15. Le Trong, I.; Wang, Z.; Hyre, D. E.; Lybrand, T. P.; Stayton, P. S.; Stenkamp, R. E., *Acta Crystallographica Section D* **2011,** *67*, 813-821.
16. Particle Tracing Module User's Guide. 6.1, C. M. v., Ed. COMSOL AB, Stockholm, Sweden: 2022.
17. Yusko, E. C.; Bruhn, B. R.; Eggenberger, O. M.; Houghtaling, J.; Rollings, R. C.; Walsh, N. C.; Nandivada, S.; Pindrus, M.; Hall, A. R.; Sept, D.; Li, J.; Kalonia, D. S.; Mayer, M., *Nature Nanotechnology* **2017,** *12*, 360-367.
18. Lowe, B. M.; Sun, K.; Zeimpekis, I.; Skylaris, C.-K.; Green, N. G., *Analyst* **2017,** *142*, 4173-4200.
19. Hideshima, S.; Nakamura, T.; Kuroiwa, S.; Osaka, T., *ECS Transactions* **2011,** *35*, 121.
